# Trust your gut: vagal nerve stimulation in humans improves reinforcement learning

**DOI:** 10.1093/braincomms/fcab039

**Published:** 2021-03-14

**Authors:** Immo Weber, Hauke Niehaus, Kristina Krause, Lena Molitor, Martin Peper, Laura Schmidt, Lukas Hakel, Lars Timmermann, Katja Menzler, Susanne Knake, Carina R Oehrn

**Affiliations:** 1 Department of Neurology, Philipps-University Marburg, 35043 Marburg, Germany; 2 Faculty of Psychology, Neuropsychology Section, Philipps-University Marburg, 35032 Marburg, Germany; 3 Faculty of Psychology, Theoretical Neuroscience Section, Philipps-University Marburg, 35032 Marburg, Germany; 4 Center for Mind, Brain and Behavior (CMBB), Philipps-University Marburg, 35032 Marburg, Germany; 5 Department of Neurology, Epilepsy Center Hessen, Philipps University, 35043 Marburg, Germany

**Keywords:** vagal nerve stimulation, reinforcement learning, extinction, cognition, decision-making

## Abstract

Whereas the effect of vagal nerve stimulation on emotional states is well established, its effect on cognitive functions is still unclear. Recent rodent studies show that vagal activation enhances reinforcement learning and neuronal dopamine release. The influence of vagal nerve stimulation on reinforcement learning in humans is still unknown. Here, we studied the effect of transcutaneous vagal nerve stimulation on reinforcement learning in eight long-standing seizure-free epilepsy patients, using a well-established forced-choice reward-based paradigm in a cross-sectional, within-subject study design. We investigated vagal nerve stimulation effects on overall accuracy using non-parametric cluster-based permutation tests. Furthermore, we modelled sub-components of the decision process using drift-diffusion modelling. We found higher accuracies in the vagal nerve stimulation condition compared to sham stimulation. Modelling suggests a stimulation-dependent increase in reward sensitivity and shift of accuracy-speed trade-offs towards maximizing rewards. Moreover, vagal nerve stimulation was associated with increased non-decision times suggesting enhanced sensory or attentional processes. No differences of starting bias were detected for both conditions. Accuracies in the extinction phase were higher in later trials of the vagal nerve stimulation condition, suggesting a perseverative effect compared to sham. Together, our results provide first evidence of causal vagal influence on human reinforcement learning and might have clinical implications for the usage of vagal stimulation in learning deficiency.

## Teaser

Weber et al. demonstrate for the first time that vagal nerve stimulation in humans enhances reinforcement learning and decelerates extinction of learned behaviour. Behavioural modelling indicates that vagal stimulation increases reward sensitivity and shifts accuracy-speed trade-offs towards maximizing rewards.

## Graphical Abstract

**Graphical Abstract:**
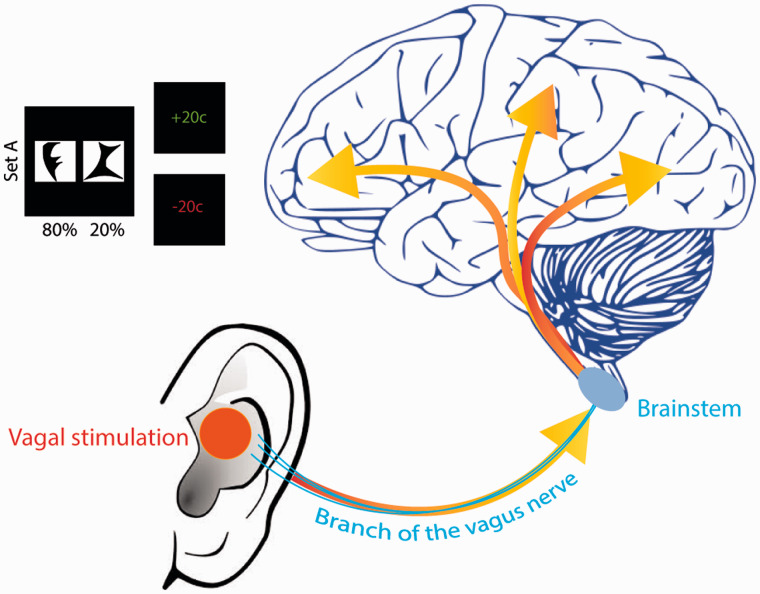

## Introduction

For decades, the vagus nerve has been described as the core of the gut-brain axis, mediating motivational and emotional states by relaying e.g. food-related information.^1^ In humans, invasive and non-invasive, i.e. transcutaneous, vagal nerve stimulation (VNS) is a common treatment for medication-resistant epilepsy and can thus be used to study causal relationships between the autonomic and the central nervous system.^2^ While there is a large body of evidence for positive effects of vagal nerve stimulation on anxiety and depression,^1^ empirical evidence suggesting a relationship between vagal nerve activity and cognitive functions is sparse. Many studies assessed effects of VNS on global measures of cognition in severely affected epilepsy patients and thus blended different cognitive functions.^2^ Other studies with Alzheimer’s and major depression patients found slight or no effects of VNS on overall cognitive function.^3^^,^^4^ The few studies that investigated VNS effects on distinct cognitive processes in epilepsy patients and healthy participants did not reveal effects on working memory,^5^ implicit learning^6^ and conflict processing.^7^ However, Clark et al.^8^ found positive effects on long-term memory formation when applying VNS during the consolidation phase of a word recognition paradigm in five epilepsy patients. A large body of evidence from animal and human studies indicates that reward processing is associated with enhanced dopamine release in the brain.^9^ Furthermore, rewarding stimulus properties leads to enhanced memory formation through interactions between dopaminergic and mnemonic brain regions.^10^

A recent study in rats demonstrated that optical stimulation of gut-innervating vagal sensory ganglions was associated with increased dopamine release from the substantia nigra and led to enhanced learning of flavour and place preferences and sustained self-stimulation behaviour.^11^ This is in line with rodent studies demonstrating enhanced cortical dopamine levels after chronic VNS^12^ and a cessation of food-related dopamine release in the midbrain after lesions of the hepatic branch of the vagus nerve.^13^ These results from animal studies suggest that vagal neurons constitute an essential component of the reward neuronal pathway. A recent study in humans indicates that healthy participants work harder for rewards during VNS compared to sham stimulation.^14^ The effect of vagal nerve stimulation on reinforcement learning in humans is still unknown. Reinforcement learning involves decision processes, which can be dissected into cognitive sub-processes using drift-diffusion models (DDM^15^). DDM decision processes based on two performance parameters, i.e. accuracy and reaction time, and constitutes one of the most widely used methods for the investigation of value-based choices.^15^ DDM disentangles decision-making into four sub-processes: (i) non-decision operations reflecting perceptual and motor computations, (ii) internal starting bias towards response options (e.g. elicited by salience) and (iii) two core parts of the decision process: (i) the drift rate: i.e. how fast the participant extracts relevant stimulus information based on previous experience and (ii) boundary separation: how much information is needed in order to make a decision.

Here, we investigate a causal relationship between vagal tone and reinforcement learning in eight long-standing seizure-free, VNS-naive epilepsy patients. In a within-subject design, we applied VNS and sham stimulation while patients performed a reward-based forced-choice learning paradigm.^16^ We applied a computerized task, which allowed us to observe effects of VNS on a trial-by-trial basis and to model behavioural sub-components of the decision process using DDM.^15^ Corresponding to the animal studies,^11^^,^^12^ we hypothesized that VNS will improve reinforcement learning and lead to a perseverance of learned behaviour.

## Methods

### Patients

Seventeen patients with temporal lobe epilepsy were recruited from the Department of Neurology at the University Hospital Marburg, Germany. We excluded patients with psychiatric comorbidities by means of the Beck Depression Inventory^17^ and the Quality of Life in Epilepsy questionnaire.^18^ As this experiment was part of a larger study investigating VNS effects on neuroimmunological, salimetric and neuropsychological measures, nine patients dropped out on one testing day due to the extensive testing pipeline. Eight patients completed the paradigm on both days (six females, five with left hemisphere epilepsy, mean age ± SD: 43.88 ± 10.93 years). Patients had been seizure-free for at least one year (mean ± SD 7.25 ± 4.50 years) and completed a neuropsychological test battery including measures of executive functioning, memory, recall and implicit memory. Furthermore, we obtained the neuroticism/extraversion/openness personality inventory by Costa und McCrae, assessing the big five personality traits^19^ and the positive and negative affect schedule^20^ (Supplementary Table 1). All patients signed written informed consent. The study was approved by the ethics committee of the University of Marburg and was conducted according to the Declaration of Helsinki.

### Paradigm

**Figure 1:**
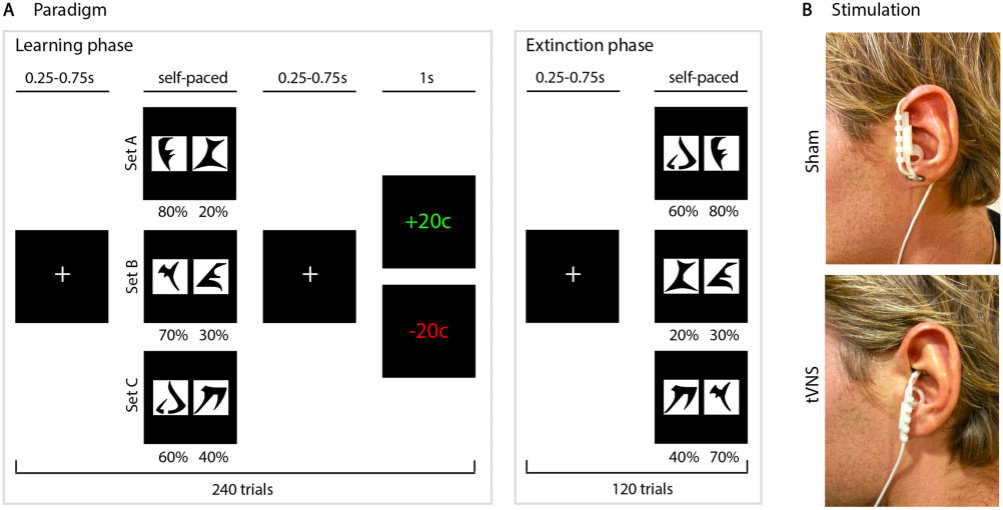
**Experimental design and electrode placement.** (**A**) The task comprised two consecutive parts: the learning and the extinction phase. During the learning phase, patients were repeatedly presented with three fixed pairs of stimuli (240 trials, 80 trials per set). The stimuli consisted of six letters from the artificial Klingon alphabet (https://www.dafont.com, last accessed: 18 Mar 2021), which were organized in three sets. Set A consisted of one stimulus with a reward probability of 80% and one with 20%, Set B with 70% and 30% and Set C with 60% and 40%, respectively. Patients were instructed to indicate as fast as possible by button press, which of both stimuli was associated with a higher reward. Pairs during the learning phase were fixed, but the side of presentation randomized for each trial. Associations between stimuli and reward probabilities were randomized for each patient. For each testing day, a different set of stimuli was used. After completion of the learning phase, an extinction phase followed, in which no feedback was given. During this phase, in each trial, a pseudo-random combination of the six previously presented letters was presented (120 trials). In total, the duration of the paradigm was ∼15 min. (**B**) Illustration of location of the stimulation electrode during the transcutaneous vagus nerve stimulation and the sham condition. In the sham condition, the probe was applied to the centre of the left lobule (top). In the active VNS condition, the probe was applied to the left cymba conchae in order to stimulate the auricular branch of the vagus nerve (bottom, image from^6^).

During stimulation, patients performed a probabilistic forced-choice learning task, in which they could earn money for correct choices^16^ (Fig. 1A). In a subsequent extinction phase, reward was omitted. Patients were seated in an acoustically shielded chamber and performed the task using a standard keyboard in front of a computer monitor. Patients received written and oral instructions and performed several practise trials. Different stimuli were used for stimulation conditions and instructions. The experimenter ensured that each patient understood the task before starting with the paradigm.

### Transcutaneous vagus nerve stimulation

In a double-blind design, VNS-naive patients received transcutaneous vagus nerve and sham stimulation on the left ear on two separate days—separated by at least two weeks—in pseudo-randomized order (Cerbomed, Nemos, Cerbomed GmbH, Erlangen). We applied VNS and sham stimulation at the same frequency (25 Hz), but at different locations (Fig. 1B). In the sham condition, the probe was applied to the centre of the left lobule. In the active VNS condition, the probe was applied to the left cymba conchae to stimulate the auricular branch of the vagus nerve according to the guidelines of the manufacturer. Stimulation amplitude was set below the average of individual sensitivity and pain threshold of each patient (mean ± SD stimulation amplitude: VNS 1.29 ± 0.61 mA; sham: 1.25 ± 0.49 mA). An independent clinician attached the stimulation device and concealed it with a headband covering the entire ear. Thus, experimenter and patients were blind to the stimulation condition.

 On both testing days, patients were continuously stimulated for 3 h before as well as during the behavioural experiment. Patients were told that the aim of the study was to compare the effects of two different stimulation sites for VNS without priming patients to expected effects at each probe position. None of the patients reported subjective differences between stimulation conditions, in particular in regard to gastrointestinal or cardiac sensations.

### Statistics

The identical analyses were performed for the study and extinction phase. We performed all calculations with Matlab 2016b (The Mathworks inc), the diffusion model analysis toolbox toolbox,^21^ the Fieldtrip toolbox^22^ and self-written code. We set the alpha level to 0.05.

#### Effects on accuracy

To analyse the time-resolved update of accuracies, we calculated the cumulative accuracies per trial according to:

(1) $$\mathrm{cACC}\left(\mathrm{trial}\right)=\frac{\sum_{i=1}^{\mathrm{trial}}\mathrm{correct}\;\mathrm{trials}}{\mathrm{trial}}.$$

We evaluated differences between the VNS and sham condition by means of non-parametric cluster-based permutation tests.^23^ This procedure ranks effect sizes in relation to a permutation distribution. The reason why we used cluster-based permutation testing is 2-fold: (i) In contrast to parametric statistical tests, permutation tests do not depend on any prior assumptions concerning the shape of the underlying distributions. This is especially important for data sets with low to medium sample sizes, where Gaussian distributions cannot be tested reliably. As such, we used permutation testing in order to test most conservatively. (ii) This procedure, originally developed for imaging studies, effectively controls for Type I errors during multiple testing.^23^ The surrogate distribution was created by randomizing condition labels (10 000 permutations).

#### Drift-diffusion modelling

**Figure 2:**
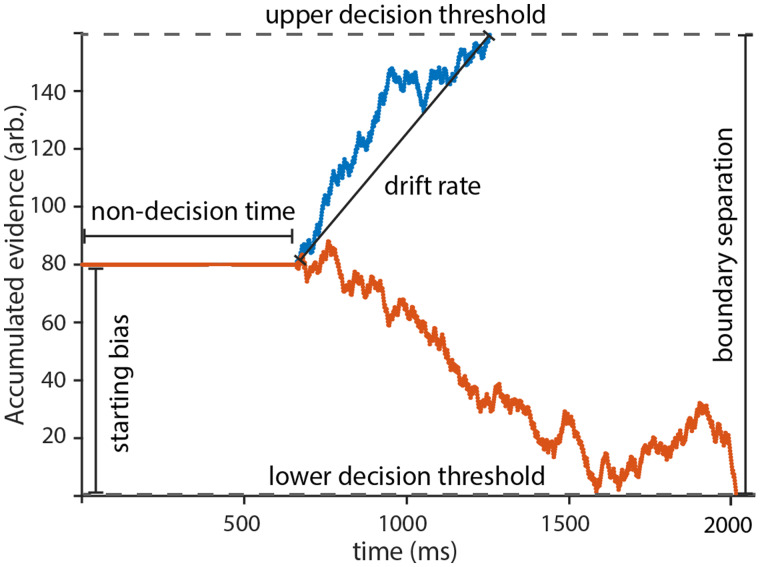
**Schematic representation of the drift and diffusion model.** One possibility to model the decision process in a two-alternative forced-choice paradigm is using the well-established drift and diffusion model. The DDM assumes that subjects slowly accumulate and integrate evidence at every time-step for one of two choices until a decision boundary is reached (dotted lines). The evidence, which constitutes sensory input and its integration with prior knowledge is assumed to be noisy and thus modelled as a stochastic process. The red and blue lines represent the accumulated evidence associated with the two opposing choices for the decision. Using the reaction time of individual subjects, the decision process may be dismantled into four parameters: non-decision time, starting bias, drift rate and boundary separation.

We modelled components of the decision process using DDM for continuous non-overlapping blocks of 30 trials using the diffusion model analysis toolbox toolbox^21^ (Fig. 2). DDM are among the most established sequential-sampling models to analyse binary forced-choice based behavioural responses. The model is based on the assumption that this decision process is preceded by a continuous cumulation of noisy evidence extracted from sensory information. This cumulation finally leads to either one of the two decision boundaries representing the possible behavioural options (see sample paths in Fig. 2). The model integrates behavioural accuracies and distributions of reaction times into four key parameters, representing specific cognitive processes. The drift rate quantifies the rate at which information, e.g. reward value, may be extracted from sensory information. High drift rates lead to faster decisions. The boundary separation describes the amount of evidence necessary to reach a decision and thus represents an accuracy-speed trade-off. An a priori favored decision is quantified by the starting bias, while the non-decision time integrates processes unrelated to the decision, such as attention or movement execution.^15^ While the DDM was originally developed to analyse simple perceptual decisions,^24^ it later became well established in studies concerned with value-based decision processes.^25^^,^^26^ First, we fitted a model, in which parameters were allowed to change freely between stimulation conditions. Second, we compared the evidence to a model with fixed parameters for both conditions using a Chi-square test, to test if any difference between stimulation conditions could be detected. If the models differed significantly, we performed a second-level analysis, to disentangle for which parameters the stimulation effect occurs. For this, we fixed all but one parameter and repeated the previous testing for all four parameters. To account for within and between subject variability we modelled the drift and diffusion process as a random effects model, with variability of drift rate, non-decision time and starting point as additional free parameters. All statistical comparisons were Bonferroni corrected by a factor blocks × parameters, resulting in an adapted alpha level of 0.002 for the learning phase and 0.003 for the extinction phase.

### Prediction of stimulation effect on accuracy

To assess the impact of patient characteristics and disease-related factors on the stimulation effect, we estimated the mutual information quotient (MIQ) between the mean accuracy differences between the stimulation conditions in the significant time window and potential predictors (i.e. age, sex, neuroticism/extraversion/openness personality inventory scores, positive and negative affect schedule scores and disease-related information).^27^ The MIQ is particularly suited for the estimation of shared information between two variables in small samples while accounting for redundant information between all predictors. We used a binning estimator and adapted bin sizes according to the Freedman–Diaconis rule.^28^ We determined significant MIQ estimates by means of a surrogate-based permutation test, where labels of response and predictor variables were randomly swapped (10 000 permutations). *P*-values were subsequently corrected for multiple comparisons using Bonferroni correction.

### Data availability

All data and code used for analysis are available upon reasonable request.

## Results

### Learning phase

#### Effect of stimulation on performance

**Figure 3:**
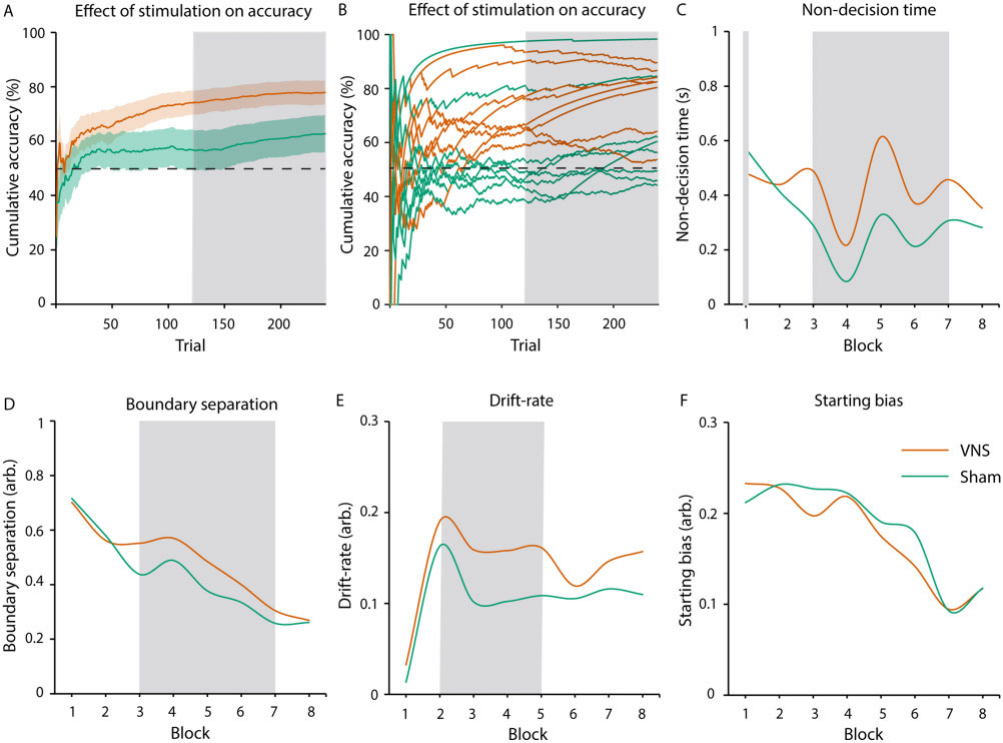
**Effect of stimulation on reinforcement learning (learning phase).** (**A** and **B**) Cluster-based permutation statistic revealed higher accuracy during the VNS compared to the sham condition (trials: 123–240, cluster statistic = 254.66, *P* = 0.04, *N* = 8 subjects, cluster-based permutation test, α = 0.05). (**A**) Average cumulative accuracies per stimulation condition. Coloured areas indicate standard errors. The dotted line indicates chance level at 50%. (**B**) Cumulative accuracies per subject and stimulation condition. (**C**–**F**) We found time (i.e. trial)—specific effects of stimulation on DDM parameters during the learning phase (model fitted over *N* = 480 trials, Chi-square test, α = 0.002, Bonferroni corrected): (**C**) non-decision time (Block 1: *P* < 0.001, Block 2: *P* = 0.017, Block 3: *P* < 0.001, Block 4: *P* < 0.001, Block 5: *P* < 0.001, Block 6: *P* < 0.001, Block 7: *P* < 0.001, Block 8: *P* = 0.024), (**D**) boundary separation (Block 1: *P* = 0.379, Block 2: *P* = 0.027, Block 3: *P* < 0.001, Block 4: *P* < 0.001, Block 5: *P* < 0.001, Block 6: *P* < 0.001, Block 7: *P* < 0.001, Block 8: *P* = 0.492), (**E**) drift rate (Block 1: *P* = 0.075, Block 2: *P* < 0.001, Block 3: *P* < 0.001, Block 4: *P* < 0.001, Block 5: *P* < 0.001, Block 6: *P* = 0.603, Block 7: *P* = 0.101, Block 8: *P* = 0.031) and (**F**) starting bias (Block 1: *P* = 0.087, Block 2: *P* = 0.044, Block 3: *P* = 0.067, Block 4: *P* = 0.755, Block 5: *P* = 0.304, Block 6: *P* = 0.003, Block 7: *P* = 0.846, Block 8: *P* = 0.959). In all subplots, shaded grey areas indicate significant differences between stimulation conditions.

Cluster statistics revealed that accuracies were significantly larger in the VNS condition than in the sham condition, which was time-specific (trial 123–240, *P* = 0.04, Fig. 3A and B, Supplementary Fig. 1). Subsequent tests against chance level demonstrated that patients effectively learned in the VNS (trials 26–240: *P* < 0.01), but not in the sham condition (no cluster found). There was no effect of stimulation order (Day 1 versus Day 2) on accuracies (largest cluster: *P* = 0.20). We did not find stimulation effects on reaction times (largest cluster: *P* = 0.33, for individual reaction times see Supplementary Table 1).

#### Drift and diffusion model

Each of the eight most liberal DDM for each trial block was significantly different from models with fixed parameters for both simulation conditions (all *P* < 0.001, for fit values see Supplementary Table 4). A second-level analysis revealed a relatively higher non-decision time (all *P* < 0.001, Blocks 1 and 3–7, Fig. 3C), boundary separation (all *P* < 0.001, Blocks 3–7, Fig. 3D) and drift rate (all *P* < 0.001, Blocks 2–5, Fig. 3E) in the VNS condition. There were no effects of stimulation on starting bias (all *P* > 0.003, Fig. 3F).

#### Prediction of stimulation effect

**Figure 4:**
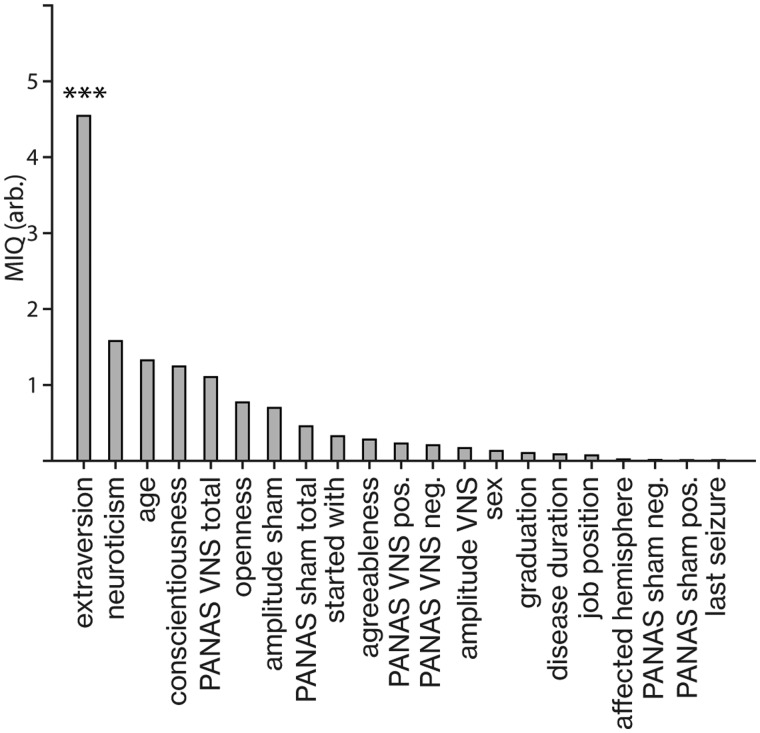
**Predictor importance of patient variables on stimulation effect (MIQ analysis).** The individual extraversion scores of patients share most predictive information with the stimulation effect on accuracy: ***P < 0.01 (MIQ = 3.90, *P* < 0.001, *N* = 8 subjects, cluster-based permutation test, Bonferroni corrected). Mutual information was calculated using a binning estimator and bin sizes optimized using the Freedman–Diaconis rule.

The analysis of MIQ revealed that the neuroticism/extraversion/openness personality inventory sub score extraversion significantly shared predictive information with the stimulation effect (MIQ = 3.90, *P* < 0.001, Fig. 4). None of the other parameters predicted the stimulation effected (all *P* > 0.11).

### Extinction phase

#### Effect of stimulation on performance

**Figure 5:**
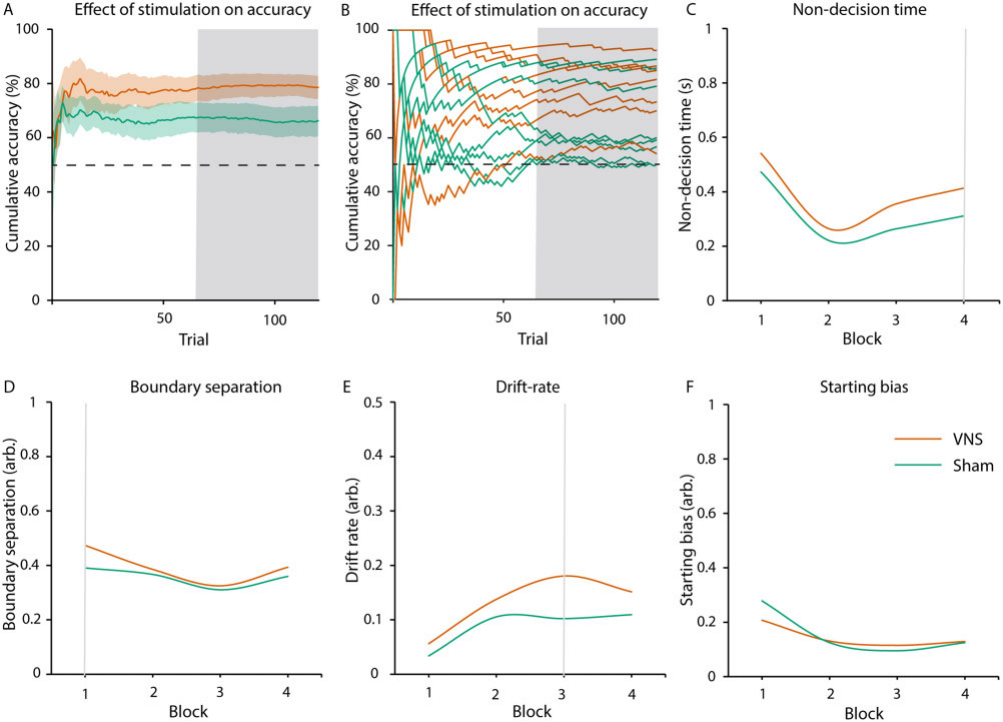
**Effect of stimulation on accuracy in extinction phase.** (**A** and **B**) Cluster-based permutation statistic shows differences between stimulation conditions on accuracy (trials 66–120, cluster statistic = 138.50, *P* = 0.02, *N* = 8 subjects, cluster-based permutation test, α = 0.05). (**A**) Average cumulative accuracies per stimulation condition. Coloured areas indicate standard errors. The dotted line indicates chance level at 50%. (**B**) Cumulative accuracies per subject and stimulation condition. We found time (i.e. trial)—specific effects of stimulation on DDM parameters during the extinction phase (model fitted over *N* = 480 trials, Chi-square test, α = 0.003, Bonferroni corrected): (**C**) non-decision time (Block 1: *P* = 0.081, Block 2: *P* = 0.238, Block 3: *P* = 0.007, Block 4: *P* = 0.001), (**D**) boundary separation (Block 1: *P* = 0.003, Block 2: *P* = 0.226, Block 3: *P* = 0.289, Block 4: *P* = 0.057), (**E**) drift rate (Block 1: *P* = 0.041, Block 2: *P* = 0.022, Block 3: *P* < 0.001, Block 4: *P* = 0.010) and (**F**) starting bias (Block 1: *P* = 0.012, Block 2: *P* = 0.616, Block 3: *P* = 0.021, Block 4: *P* = 0.689). In all subplots, grey-shaded areas indicate significant differences between conditions. To account for within and between subject variability, we modelled the drift and diffusion process as a random effects model, with variability of drift rate, non-decision time and starting point as free parameters.

Cluster statistics showed that accuracies were higher for VNS in comparison to the sham condition. This effect was specific for later trials of the extinction phase (Trials 66–120: *P* = 0.02, Fig. 5A and B, Supplementary Fig. 2), indicating a perseverative effect of stimulation on learning. There was no effect of stimulation order on accuracy (no cluster found) or reaction times (no cluster found).

#### Drift and diffusion model

Blocks 1, 3 and 4 of the most liberal DDM yielded significant differences between stimulation conditions (Block 1: *P* = 0.01, Block 2: *P* = 0.25, Block 3: *P* < 0.001, Block 4: *P* < 0.01, fit values: See Supplementary Table 5). Analogous to the learning phase, we found stimulation effects on non-decision time (*P* = 0.001, Block 4, Fig. 5C), boundary separation (*P* = 0.003, Block 1, Fig. 5D) and drift rate (*P* < 0.001, Block 4, Fig. 5E). There was no effect on starting bias (all *P* > 0.012, Fig. 5F).

#### Effects of stimulation on mood and other bodily sensations

Wilcoxon signed-rank tests revealed no differences of positive, negative or total positive and negative affect schedule scores between both stimulation conditions [VNS versus sham: positive/VNS 16.25 ± 2.12 (mean ± standard error), positive/sham 17.38 ± 1.56, *P* = 0.16; negative/VNS: 17.25 ± 1.32, negative/sham: 17.75 ± 1.33, *P* = 0.71; total/VNS: 33.50 ± 3.41, total/sham: 35.13 ± 2.66, *P* = 0.38, Wilcoxon signed-rank test, Bonferroni corrected].

## Discussion

In this study, we show for the first time that VNS in humans positively influences reinforcement learning. During the learning phase, VNS was associated with a higher overall accuracy of decisions (Fig. 3A and B) than during sham stimulation. The effect was time specific, i.e. it appeared after several trials of learning (123 trials). After omission of reward, VNS led to a perseverance of learned behaviour. This effect occurred in the second half of the extinction phase, when accuracy had dropped during sham stimulation, but not VNS.

These results are in accordance with a previous study in rats demonstrating that optical stimulation of vagal sensory neurons was associated with enhanced reinforcement learning and perseverance of behaviour, analogous to the decelerated extinction in our study.^11^ Along with other results in rats,^12^ this rodent study indicates that vagal stimulation is associated with enhanced dopamine release in the brain. It is well established that dopamine release encodes prediction errors—the discrepancy between expected and observed reward—and is the core neurotransmitter involved in reinforcement learning.^9^ Thus, one could speculate that enhanced dopamine release by vagal stimulation constitutes the underlying neural mechanism of the effects of VNS on reinforcement learning observed in this study. However, here, we do not explicitly demonstrate the role of dopamine and it is very likely that VNS additionally activates other transmitter systems. Several studies demonstrated that VNS increases serotonin in the animal^12^ and norepinephrine levels in the human brain,^29^ both of which are associated with reward expectation.^30^^,^^31^

Furthermore, we investigated the effects of VNS on sub-components of reward-based decision-making. We found effects of VNS on boundary separation and drift rates (Fig. 3D and E). In reward-based paradigms, drift rate can be interpreted as a proxy of reward sensitivity.^15^ In addition, VNS raised the internal decision threshold, favouring accuracy over speed in comparison to sham stimulation (boundary separation, Fig. 3D). Based on the assumption that vagal stimulation promotes dopaminergic activity,^11^ our results are in line with previous studies demonstrating a positive correlation of dopaminergic activity and reward sensitivity in animal^32^ and human studies.^33^

We found that VNS during learning did not only enhance processes linked to decision-making but also increased behavioural components tied to sensory and attention processes (non-decision time, Fig. 3C). This result may be linked to previous reports of increased post-error slowing during vagal stimulation in humans (i.e. slowing of the subsequent response^34^). Other VNS studies reported an increase of attentional processes, which may bind cognitive resources during decision-making.^35^ This explanation is further supported by effectiveness of dopamine-releasing drugs for patients with attention-deficit disorder.^36^ Stimulation did not affect internal preference for either response (starting bias, Fig. 3F), which demonstrates the specificity of the stimulation effects on the latter three aspects of decision-making.

Mutual information analysis revealed that no patient characteristics or disease-related factors impacted the stimulation effect on reinforcement learning. We found a selective relationship between extraversion of patients and the extent of the stimulation effects. A supplementary analysis indicates that patients with relatively lower extraversion profited most from stimulation, i.e. the stimulation effect in patients deviated during sham, but not VNS stimulation (Supplementary Fig. 3). These results are in accordance with previous studies showing that individual differences in extraversion predict reward sensitivity in humans.^37^ However, this finding should be interpreted with caution due to the small sample size and should be corroborated in future studies. As different affective states during stimulation may also potentially influence behavioural results, we also included positive and negative affect schedule scores recorded during both stimulation conditions as predictors for our mutual information analysis. However, neither positive, negative nor total scores per condition had a significant contribution to predicting the influence of stimulation on learning accuracy. In addition, there were no self-reports on gastrointestinal, cardiac or other sensations during either of both stimulation conditions. Overall, this indicates that the stimulation effect is not indirectly mediated by mood.

One limitation of our study is the small simple size. However, we show consistent results across patients. We analysed the data conservatively by using non-parametric statistics and corrected rigorously Type I errors were applicable. A second drawback of our study is the study population. Due to ethical reasons, we refrained from applying VNS to healthy participants. However, to approximate effects in the healthy population and make inferences more generalizable, we exclusively included long-standing (i.e. minimum of 1 year) seizure-free patients without neuropsychological deficits.

In summary, we demonstrate for the first time that vagal nerve stimulation in humans enhances reinforcement learning and decelerates extinction of learned behaviour. Behavioural modelling indicates that vagal tone increases reward sensitivity and shifts accuracy-speed trade-offs towards maximizing rewards.

## Supplementary material

Supplementary material is available at *Brain Communications* online.

## Supplementary Material

fcab039_Supplementary_Data

## Abbreviations

DDM =: drift-diffusion model
MIQ =: mutual information quotient
VNS =: vagal nerve stimulation
