# Supplementary material for: Trust your gut: vagal nerve stimulation in humans improves reinforcement learning
Source: Brain Commun. 2021 Mar 14;3(2):fcab039. doi: 10.1093/braincomms/fcab039 (PMC8066886; doi:10.1093/braincomms/fcab039)
Supplement: fcab039_Supplementary_Data [file fcab039_supplementary_data.zip › Supplementary_material.docx]

# Supplementary material

**Supplementary Table 1: Patient data**

| **ID** | | | **1** | **2** | **3** | **4** | **5** | **6** | **7** | **8** |
| --- | --- | --- | --- | --- | --- | --- | --- | --- | --- | --- |
| **Sex** | | | f | m | f | f | f | f | f | m |
| **Age** | | | 55 | 53 | 53 | 32 | 42 | 41 | 25 | 50 |
| **Disease duration** | | | 42 | 22 | 25 | 17 | 33 | 28 | 9 | 6 |
| **Affected hemisphere** | | | right | left | right | left | left | left | left | right |
| **Years since last seizure (YLS)** | | | 1 | 16 | 5 | 9 | 5 | 10 | 5 | 7 |
| **Stimulation** | **VNS** | | 2.5 | 1 | 0.8 | 0.9 | 1.5 | 1.8 | 1 | 0.8 |
| **amplitude (mA)** | **Sham** | | 1 | 0.7 | 1.2 | 2.2 | 0.8 | 1.7 | 1.2 | 1.2 |
| **Started with (SW)** | | | Sham | ON | ON | Sham | Sham | ON | ON | Sham |
| **Graduation** | | | 1 | 3 | 3 | 3 | 3 | 2 | 3 | 1 |
| **Highest job position** | | | 2 | 4 | 2 | 2 | 3 | 2 | 2 | 1 |
| **WAIS-IV** | **MZ_RW** | | 15 | 24 | 20 | 18 | 25 | 22 | 24 | 12 |
|  | **MZ_WP** | | 9 | 14 | 10 | 8 | 15 | 11 | 12 | 6 |
| **Socio-demographic** | **Verbal-IQ (VIQ)** | | 96 | 126 | 109 | 107 | 122 | 101 | 119 | 94 |
| **estimation scale** | **Total IQ (TIQ)** | | 99 | 115 | 109 | 109 | 124 | 103 | 118 | 93 |
| **NEO-PI** | **Neuroticism (Neu)** | | 3.92 | 3.06 | 2.77 | 3.21 | 2.81 | 3.02 | 3.56 | 3.27 |
|  | **Extraversion (Ext)** | | 3.13 | 3.29 | 2.81 | 3.46 | 3.67 | 3.35 | 3.25 | 3.5 |
|  | **Openness (Ope)** | | 3.79 | 3.35 | 2.98 | 3.48 | 3.73 | 3.69 | 4.04 | 3.69 |
|  | **Agreeableness (Agr)** | | 3.58 | 3.63 | 3.46 | 3.94 | 3.65 | 3.71 | 3.71 | 3.83 |
|  | **Conscientiousness (Con)** | | 3.42 | 3.56 | 3.54 | 3.52 | 3.71 | 3.69 | 3.96 | 3.96 |
| **PANAS** | **Positive** | **VNS** | 12 | 25 | 10 | 25 | 10 | 16 | 15 | 17 |
|  | **Negative** | **VNS** | 14 | 22 | 14 | 23 | 13 | 18 | 18 | 16 |
|  | **Total** | **VNS** | 26 | 47 | 24 | 48 | 23 | 34 | 33 | 33 |
|  | **Positive** | **Sham** | 14 | 27 | 13 | 18 | 14 | 17 | 18 | 18 |
|  | **Negative** | **Sham** | 16 | 26 | 19 | 17 | 16 | 19 | 14 | 15 |
|  | **Total** | **Sham** | 30 | 53 | 32 | 35 | 30 | 36 | 32 | 33 |
| **Reaction times (s)** | **Learning phase** | **VNS** | 1.79 | 1.32 | 1.59 | 2.2 | 3.49 | 1.36 | 1.47 | 1.12 |
|  |  | **Sham** | 1.66 | 1.34 | 1.69 | 1.04 | 2.42 | 0.81 | 1.65 | 1.21 |
|  | **Extinction phase** | **VNS** | 1.64 | 1.41 | 1.4 | 2.08 | 1.98 | 1.28 | 1.5 | 0.85 |
|  |  | **Sham** | 1.19 | 0.96 | 1.54 | 1.09 | 1.92 | 1.2 | 1.49 | 1.23 |

f: female, m: male; graduation: 1=no graduation, 2= secondary school, 3= technical diploma/general higher education entrance qualification; highest job position: 0= no job, 1=trainee, 2= office worker/civil servants, 3= academics/students, 4= senior staff/senior officer, WAIS-IV: Wechsler Adult Intelligence Scale, MZ_RW: raw values of matrix reasoning test, MZ_WP: point values of matrix reasoning test; PANAS: positive and negative affect schedule stimulation amplitude was not different between conditions (p=0.90, two-sided paired t-test).

**Supplementary Table 2: Cumulative accuracy values averaged per block (learning phase)**

|  | Condition | VNS | | | | | | | |
| --- | --- | --- | --- | --- | --- | --- | --- | --- | --- |
|  | Block | 1 | 2 | 3 | 4 | 5 | 6 | 7 | 8 |
| ID | **1** | 61.14 | 53.39 | 55.58 | 58.39 | 59.72 | 60.29 | 62.84 | 64.17 |
|  | **2** | 70.34 | 90.87 | 94.63 | 95.16 | 93.67 | 93.11 | 91.99 | 90.71 |
|  | **3** | 81.65 | 83.92 | 87.49 | 89.46 | 89.88 | 90.17 | 89.50 | 87.40 |
|  | **4** | 39.43 | 39.36 | 55.19 | 64.95 | 69.64 | 75.16 | 78.99 | 81.74 |
|  | **5** | 49.03 | 57.45 | 64.90 | 73.73 | 76.55 | 78.79 | 81.50 | 82.30 |
|  | **6** | 60.10 | 68.39 | 63.89 | 62.94 | 66.52 | 72.13 | 76.39 | 79.13 |
|  | **7** | 47.21 | 62.70 | 71.27 | 76.08 | 78.89 | 80.65 | 81.47 | 83.37 |
|  | **8** | 48.67 | 68.99 | 67.24 | 65.46 | 63.11 | 58.87 | 56.73 | 53.22 |
|  | Condition | Sham | | | | | | | |
|  | Block | 1 | 2 | 3 | 4 | 5 | 6 | 7 | 8 |
| ID | **1** | 40.99 | 52.59 | 53.13 | 51.20 | 48.58 | 48.20 | 48.25 | 46.50 |
|  | **2** | 17.78 | 50.32 | 50.72 | 52.51 | 53.31 | 55.32 | 56.18 | 56.89 |
|  | **3** | 41.89 | 44.78 | 48.42 | 48.15 | 49.85 | 55.63 | 58.84 | 60.84 |
|  | **4** | 43.12 | 37.99 | 36.88 | 38.30 | 38.61 | 41.01 | 41.50 | 43.89 |
|  | **5** | 77.13 | 74.52 | 77.41 | 79.86 | 79.64 | 81.07 | 82.63 | 84.19 |
|  | **6** | 71.16 | 93.15 | 95.97 | 97.14 | 97.78 | 97.87 | 97.95 | 98.22 |
|  | **7** | 52.92 | 47.11 | 45.46 | 45.71 | 40.93 | 42.72 | 51.31 | 57.81 |
|  | **8** | 41.36 | 49.82 | 44.15 | 45.21 | 45.38 | 48.65 | 49.82 | 49.33 |

Each block consists of n=30 trials.

**Supplementary Table 3: Cumulative accuracy values averaged per block (extinction phase)**

|  | Condition | VNS | | | | Sham | | | |
| --- | --- | --- | --- | --- | --- | --- | --- | --- | --- |
|  | Block | 1 | 2 | 3 | 4 | 1 | 2 | 3 | 4 |
| ID | **1** | 50.44 | 70.81 | 74.11 | 73.91 | 45.96 | 58.94 | 59.56 | 56.53 |
|  | **2** | 56.90 | 65.76 | 68.27 | 70.41 | 64.39 | 70.48 | 60.54 | 59.43 |
|  | **3** | 97.33 | 89.99 | 88.70 | 86.68 | 63.90 | 48.71 | 51.50 | 51.34 |
|  | **4** | 87.54 | 91.71 | 86.33 | 86.64 | 57.55 | 53.71 | 59.04 | 58.98 |
|  | **5** | 97.40 | 93.99 | 94.24 | 93.00 | 86.18 | 85.81 | 87.39 | 85.49 |
|  | **6** | 93.04 | 77.02 | 78.21 | 80.22 | 83.26 | 87.02 | 87.30 | 88.37 |
|  | **7** | 83.47 | 80.49 | 85.01 | 85.96 | 88.95 | 80.08 | 81.15 | 78.61 |
|  | **8** | 37.14 | 47.81 | 54.15 | 56.40 | 49.45 | 44.71 | 51.97 | 49.63 |

Each block consists of n=30 trials.

**Supplementary Table 4: Badness-of-fit values for learning phase**

|  | Phase | Learning | | | | | | | |
| --- | --- | --- | --- | --- | --- | --- | --- | --- | --- |
|  | Block | 1 | 2 | 3 | 4 | 5 | 6 | 7 | 8 |
| Fitvalue | No stim. Effect | 911.51 | 1079.25 | 1182.30 | 1308.13 | 1353.99 | 1242.53 | 1396.77 | 1469.83 |
|  | Stim. Effect | 851.51 | 1031.24 | 1115.00 | 1253.46 | 1278.71 | 1209.25 | 1353.08 | 1445.12 |

Shown are the badness-of-fit values of the DDMs per trial block during the learning phase. Stim. Effect: all DDM-parameters were allowed to vary during stimulation condition, No stim. Effect: fixed parameters. The lower the values, the better is the modelfit.

**Supplementary Table 5: Badness-of-fit values for extinction phase**

|  | Phase | Extinction | | | |
| --- | --- | --- | --- | --- | --- |
|  | Block | 1 | 2 | 3 | 4 |
| Fitvalue | No stim. Effect | 1137.44 | 1237.45 | 1249.21 | 1328.56 |
|  | Stim. Effect | 1115.76 | 1223.98 | 1215.40 | 1302.38 |

Shown are the badness-of-fit values of the DDMs per trial block during the extinction phase. Stim. Effect: all DDM-parameters were allowed to vary during stimulation condition, No stim. Effect: fixed parameters. The lower the values, the better is the model fit.


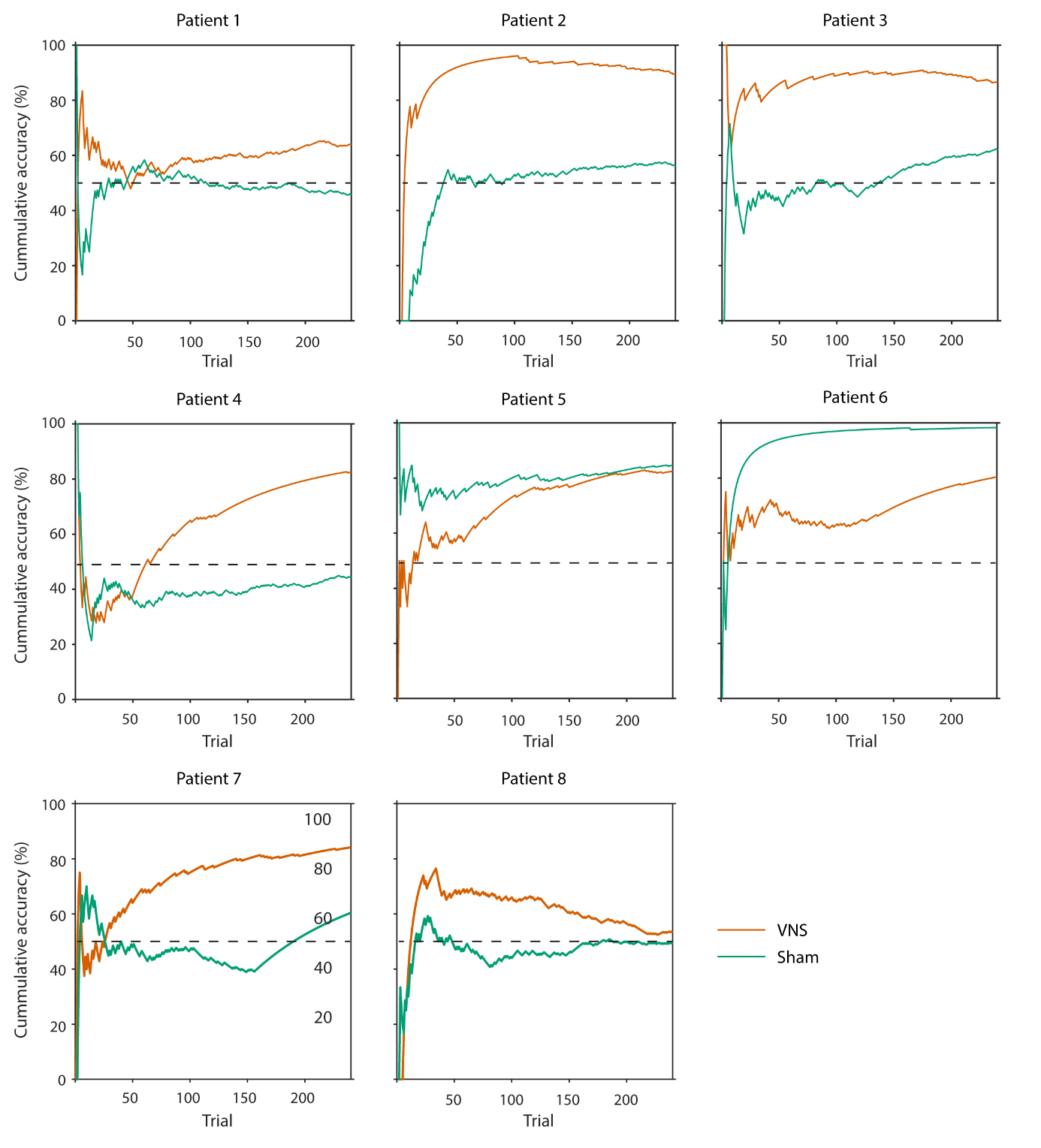


**Supplementary Figure 1: Individual cumulative accuracies during learning phase for each patient per condition.** Dotted lines represent chance level at 50%.


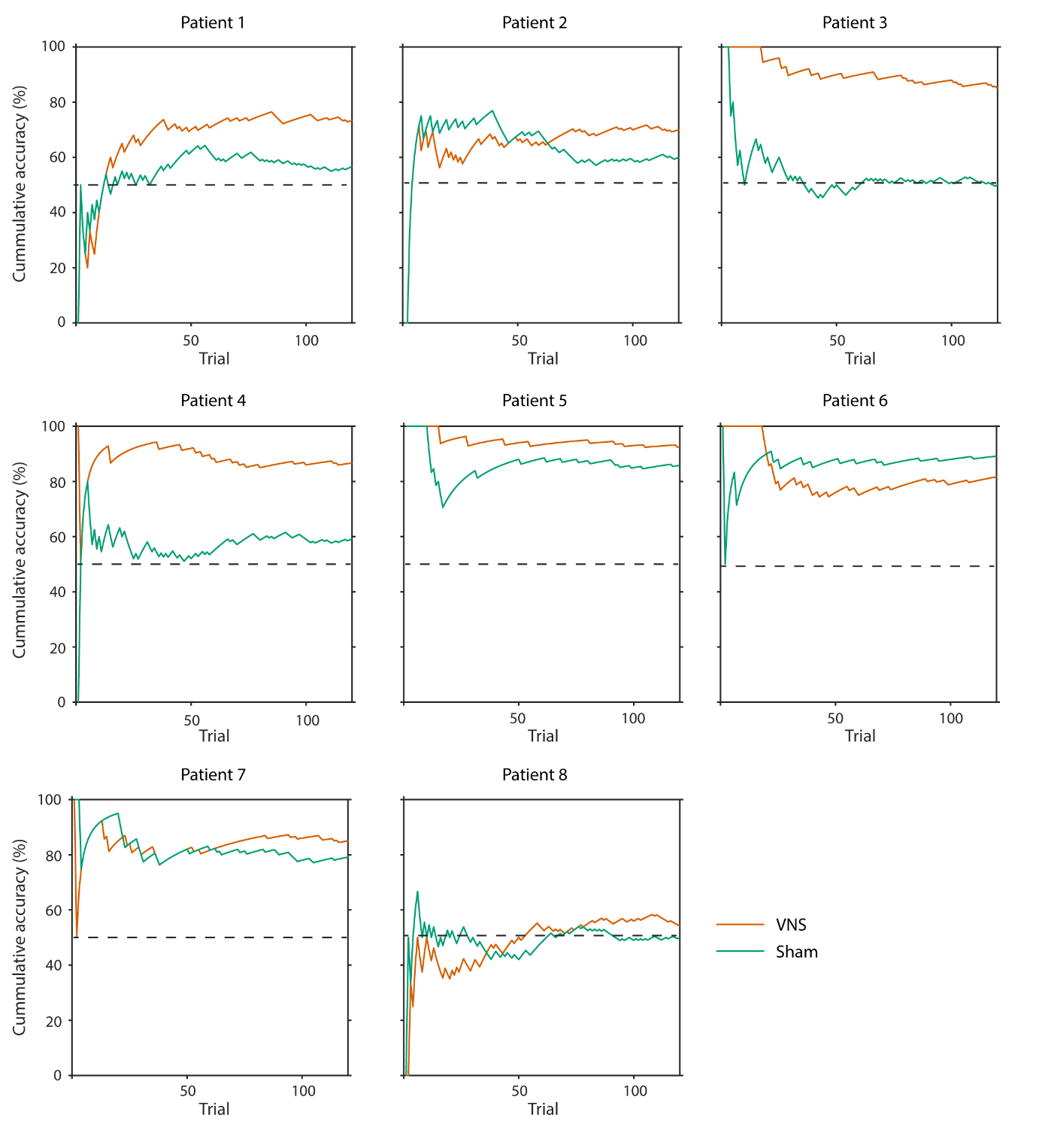


**Supplementary Figure 2: Individual cumulative accuracies during extinction phase for each patient per condition.** Dotted lines represent chance level at 50%.


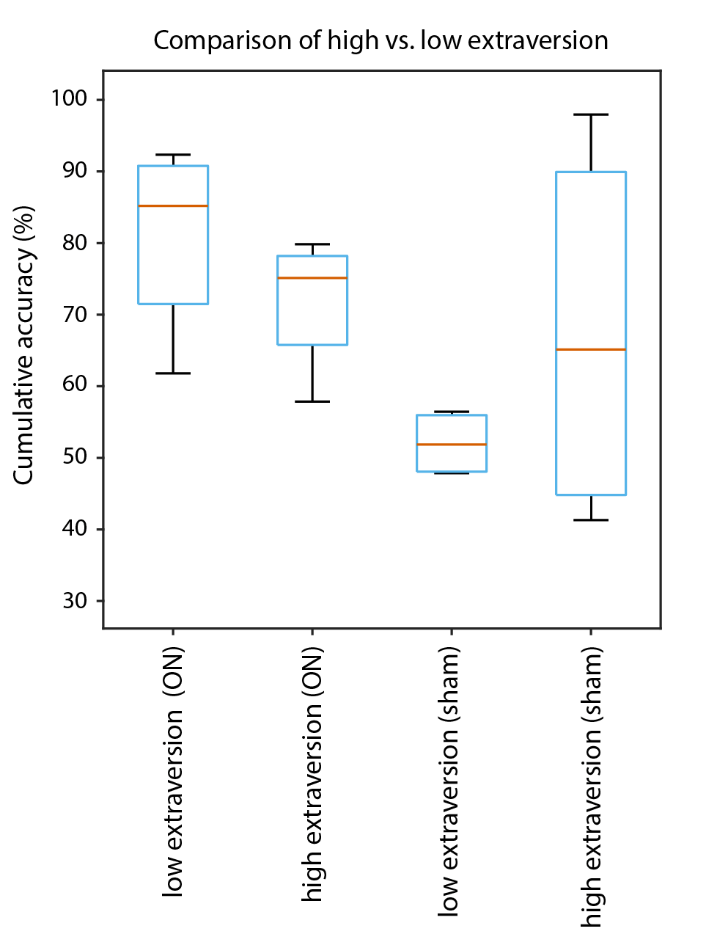


**Supplementary Figure 3: Relationship of accuracies for low vs. high extraversion and both stimulation conditions (n=4 per group).** The upper and lower edges of the boxes indicate the 25^th^ and 75^th^ percentile, respectively. Red lines indicate the median and whiskers the most extreme values.
