# Supplementary material for: Trust your gut: vagal nerve stimulation in humans improves reinforcement learning
Source: Brain Commun. 2021 Mar 14;3(2):fcab039. doi: 10.1093/braincomms/fcab039 (PMC8066886; doi:10.1093/braincomms/fcab039)
Supplement: fcab039_Supplementary_Data [file fcab039_supplementary_data.zip › Review_files.zip!/Original Submission-Authors Response.pdf]

RESPONSE TO REVIEWERS

Brain Communications, Ref: Submission ID BRAINCOM-2020-251

Reviewer #1 and Reviewer #2

1. **Reviewer 1:** One critical aspect that is unclear refers to how exactly the sham condition was defined. Did the subjects detect any sensations upon vagal stimulation? How did the authors reassure themselves that subjects could not discriminate between sham vs vagal conditions? Please explain.

   Reviewer 2: However my stimulation was lessened by the lack of details regarding what would be the control condition. It is unclear if subjects were aware of stimulation and if so how this was controlled for. Just the perception of sensory stimulation may have driven subjects to focus and improve performance in response to the experimental setting.

Response: We apologize for the lack of clarity in our manuscript. The sham condition was defined as stimulation at the same frequency (25 Hz) and amplitude (mean±SD: VNS 1.29±0.61 mA; sham: 1.25±0.49 mA) at a different stimulation site. In the sham condition, the probe was applied to the center of the left lobule (Figure 1B, top). In the active VNS condition, the probe was applied to the left cymba conchae in order to stimulate the auricular branch of the vagus nerve according to the guidelines of the manufacturer (Figure 1B, bottom). An independent clinician attached the stimulation device and concealed it with a headband covering the entire ear. Thus experimenter and patients were blind to the stimulation condition.

Importantly, patients were blind to the research question. We told all participants, that the aim of the study was to compare two different stimulation sites for VNS without priming patients to expected effects at each probe position. Further, none of the patients reported any subjective differences between stimulation conditions, in particular in regard to gastrointestinal, cardiac or any other sensory sensations. Therefore, we do not expect any systematic bias regarding stimulation settings influencing our results. We added a detailed description on the stimulation settings to the methods section of the manuscript:

Methods, p. 06, sub-section “2.3. Transcutaneous vagus nerve stimulation”:

“In a double-blind design, VNS-naive patients received transcutaneous vagus nerve and sham stimulation on the left ear on two separate days - separated by at least two weeks - in pseudo-randomized order (Cerbomed, Nemos, Cerbomed GmbH, Erlangen). We applied VNS and sham stimulation at the same frequency (25 Hz), but at different locations (Fig. 1B). In the sham condition, the probe was applied to the center of the left lobule. In the active VNS condition, the probe was applied to the left cymba conchae in order to stimulate the auricular branch of the vagus nerve according to the guidelines of the manufacturer. Stimulation amplitude was set below the average of individual sensitivity and pain threshold of each patient (mean±SD stimulation amplitude: VNS 1.29±0.61 mA;

sham: 1.25±0.49 mA). An independent clinician attached the stimulation device and concealed it with a headband covering the entire ear. Thus, experimenter and patients were blind to the stimulation condition.

Patients were continuously stimulated for three hours before and during the paradigm and were told that the aim of the study was to compare effects of two different stimulation sites for VNS without priming patients to expected effects at each probe position. None of the patients reported subjective differences between stimulation conditions, in particular in regard to gastrointestinal or cardiac sensations."

2. **Reviewer 1:** More generally did the subjects report any detectable changes in mood or physiological variables such as gastrointestinal or cardiac tone? The "reward" effect was purely task-dependent?

   Reviewer 2: Also how about self reports on subjective feelings such as changes in mood, gastrointestinal sensations, and so on? I believe that without these basic type of information it is difficult to assess the potential quality of the manuscript.

<u>Response:</u> We thank the reviewers for this very important point. In order to assure that effects on reward processing were mediated indirectly via changes in mood, we quantified mood using the positive and negative affect schedule (PANAS). We did not find any effects of stimulation condition (tVNS vs. sham) on positive, negative or total PANAS scores and now report these findings in the revised version of the manuscript:

<u>Results, p. 11, sub-section "Effects of stimulation on mood and other bodily sensations":</u>

"*Wilcoxon-signed-rank tests revealed no differences of positive, negative or total PANAS scores between both stimulation conditions (VNS vs. sham: positive/VNS 16.25±2.12 [mean±standard error], positive/sham 17.38±1.56, p= 0.16; negative/VNS: 17.25±1.32, negative/sham: 17.75±1.33, p =0.71; total/VNS: 33.50±3.41, total/sham: 35.13±2.66, p=0.38, Wilcoxon-signed-rank test, Bonferroni corrected)."*

Further, inclusion of individual PANAS scores into our mutual information analysis did not reveal any significant impact on the stimulation effect on reward learning. We adapted Figure 4 to include PANAS scores. We also included PANAS in the methods section:

<u>Methods, p. 05, sub-section 2.1. "Patients":</u>

"Further, we obtained the NEO Personality Inventory by Costa und McCrae (NEO-PI), assessing the big five personality traits (Ostendorf and Angleitner, 2004) and the positive and negative affect schedule (PANAS, Breyer and Bluemke, 2016, Supplementary Tab. S1)."

Methods, p. 09, sub-section "Prediction of stimulation effect on accuracy":

"In order to assess the impact of patient characteristics and disease-related factors on the stimulation effect, we estimated the mutual information quotient (MIQ) between the mean accuracy differences between the stimulation conditions in the significant time window and potential predictors (i.e. age, sex, NEO-PI scores, PANAS scores and disease-related information) (Gulgezen et al., 2009)."

Further, we added the following sentence to the methods section:

Methods, p. 06, sub-section 2.3. "2.3. Transcutaneous vagus nerve stimulation":

"None of the patients reported subjective differences between stimulation conditions, in particular in regard to gastrointestinal or cardiac sensations."

We added the following paragraph to the discussion (p. 13):

"As different affective states during stimulation may also potentially influence behavioral results, we also included PANAS scores recorded during both stimulation conditions as predictors for our mutual information analysis. However, neither, positive, negative nor total scores per condition had a significant contribution to predicting the influence of stimulation on learning accuracy. Additionally, there were no self-reports on gastrointestinal, cardiac or other sensations during any of both stimulation conditions. Overall, this indicates that the stimulation effect is not indirectly mediated by mood."

Reviewer #1

3. **A)** Another important point that requires clarification is the choice of non-parametric cluster analyses; **B)** the manuscript should also include a more in-depth description, preferably not excessively technical, of drift-diffusion modeling. **C)** In general the data presented is based on model fitting and would be more clarifying to see more of the raw data associated with the subjects' behaviors.

Response: **A)** We apologize for being unclear regarding our motivation for using cluster-based permutation statistics. We added the following paragraph to the methods section:

Methods, p. 07, sub-section "Effects on accuracy":

"The reason why we used cluster-based permutation testing is two-fold: 1) In contrast to parametric statistical tests, permutation tests do not depend on any prior assumptions concerning the shape of the underlying distributions. This is especially important for data sets with low to medium sample sizes, where Gaussian distributions cannot be tested reliably. As such, we used permutation testing in order to test most conservatively. 2) This procedure, originally developed for imaging studies, effectively controls for type I errors during multiple testing (Maris and Oostenveld, 2007)."

B) We added a more in-depth description to drift-diffusion modelling to the methods section. In addition, we moved supplementary figure S1, which describes the DDM, into the main article as figure 2.

Methods, pp. 07-08, sub-section "Drift-diffusion modelling":

"DDM are among the most established sequential-sampling models to analyze binary forced-choice based behavioral responses. The model is based on the assumption, that this decision process is preceded by a continuous cumulation of noisy evidence extracted from sensory information. This cumulation finally leads to either one of two decision boundaries representing the possible behavioural options (see sample paths in Fig. 2). The model integrates behavioral accuracies and distributions of reaction times into four key parameters, representing specific cognitive processes. The drift rate quantifies the rate at which information, e.g. reward value, may be extracted from sensory information. High drift-rates lead to faster decisions. The boundary separation describes the amount of evidence necessary to reach a decision and thus represents an accuracy-speed tradeoff. An a priori favored decision is quantified by the starting bias, while the non-decision time integrates processes unrelated to the decision, such as attention or movement execution. While the DDM was originally developed to analyze simple perceptual decisions (Ratcliff, 1978), it later became well established in studies concerned with value-based decision processes (Busemeyer and Townsend, 1993; Roe et al., 2001)."

C) We apologize that results are in parts not clear enough. While a part of the study is indeed based on model-fitting, our main results (Fig 3A) are based on raw data, as illustrated in Figure 3 A. Individual behavioural data is supplied in the supplementary figures S1 and S2. The modelling analysis was performed with data of eight and four blocks each with 30 trials for the learning and extinction phase, respectively. In order to facilitate interpretation of this part, we now include two supplementary tables (S1 and S2) with averaged raw accuracy values per block for the learning and extinction phase, respectively.

Editorial Office Comments:

1. For each statistical test, please report in the figure legend, the N, test used, relevant test statistic, and exact p values (for example F or t values for ANOVAs and t-tests). Please provide details of the statistical tests in the figure legends.

   Response: We adapted legends of figures 3, 4 and 5 accordingly. For the drift-diffusion models, we previously reported p-values after Bonferroni correction in combination with the original alpha-level. Instead, we now report the original p-values in combination with the adapted alpha-levels. We adapted the methods section accordingly.

   Methods, p.08, sub-section "Drift-diffusion modelling":

*“All statistical comparisons were Bonferroni corrected by a factor blocks*parameters, resulting in an adapted alpha-level of 0.002 for the learning phase and 0.003 for the extinction phase.”*

2. To comply with our policies for rigour and reproducibility, please provide details in each figure legend of the experimental unit (N= trials, subjects). Please note our policy on pseudoreplication, we do not accept analyses where individual trials are the experimental unit when there is likely biological variability is between subjects.

Response: We added the experimental units to figure legends of figures 3, 4 and 5. While the main results, i.e. the cluster-based permutation analysis, as well as the mutual information analysis was calculated over subjects (Fig. 3A, 4 and 5A), modelling was performed over cumulated trials as to robustly estimate parameters. Inter-subject variability was accounted for by estimating models as random effects, with intra- and inter-trial variability as additional free parameters. We added the following paragraph to the methods section.

Methods, p. 08, sub-section “Drift-diffusion modelling”:

“*To account for within and between subject variability, we modelled the drift and diffusion process as a random effects model, with variability of drift rate, non-decision time and starting point as additional free parameters.”*

3. Please supply an abbreviated summary of up to 50 words (no abbreviations except gene names). This will be used in the table of contents alongside the graphical abstract. Please upload as a separate word document starting with “First author et al.…”.

Response: We added the separate document as requested.

Additional Comment:

On page 10, section 3.2. “Extinction phase”, we also included a subheading for the effects of stimulation on performance of the extinction phase, similar to the subheading of the learning phase.
