# Supplementary material for: Trust your gut: vagal nerve stimulation in humans improves reinforcement learning
Source: Brain Commun. 2021 Mar 14;3(2):fcab039. doi: 10.1093/braincomms/fcab039 (PMC8066886; doi:10.1093/braincomms/fcab039)
Supplement: fcab039_Supplementary_Data [file fcab039_supplementary_data.zip › Review_files.zip!/Original Submission-Decision Letter.pdf]

Original Submission-Decision Letter

13-Oct-2020

Dear Dr. Weber,

Thank you for your recent submission BRAINCOM-2020-251 entitled "Trust your gut: vagal nerve stimulation in humans improves reinforcement learning" to Brain Communications.

Your manuscript has been reviewed, and while the reviewers and editorial team appreciate many aspects of the paper, they raise some concerns that you will need to address in order for the paper to be considered for publication.

If you can address these concerns, I invite you to submit a revised manuscript and a response to each of the reviewers' comments. The reviewer(s)' comments are included at the bottom of this message.

In addition to the reviewers' comments, please note our editorial comments added below to comply with our requirements for presentation of scientific data at Brain Communications.

Please format your revised manuscript according to our Instructions to Authors https://academic.oup.com/braincomms/pages/General_Instructions – a summary of key points is attached to help with this.

To revise your manuscript, click the link below or log into https://mc.manuscriptcentral.com/braincom and enter your Author Centre, where you will find your manuscript title listed under "Manuscripts with Decisions." Under "Actions," click on "Create a Revision." Your manuscript number has been appended to denote a revision.

*** PLEASE NOTE: This is a two-step process. After clicking on the link, you will be directed to a webpage to confirm. ***

https://mc.manuscriptcentral.com/braincom?URL_MASK=c076d73c4e224b879c3a7ac113765233

Please upload a revised manuscript with changes tracked with track changes or in coloured text.

When submitting your revised manuscript, you will be asked to respond to the reviewer(s) comments. Please be as specific as possible in your response to all of the comments.

The editorial team at Brain Communications aims to increase transparency in scientific publishing and to demonstrate the value that peer review brings to the scientific process. Please therefore consider whether you are willing to have reviewer(s)' comments on your paper and your response published as supplementary data with the paper if it is accepted. This is entirely optional and will only be done if both the authors and reviewers agree. While submitting your revision there will be an opportunity to review or update the answer you gave previously, if necessary.

IMPORTANT: Your original files are available to you when you upload your revised manuscript. Please delete any redundant files before completing the submission.

Because we are trying to facilitate timely publication of manuscripts submitted to Brain Communications, we would appreciate your revised manuscript as soon as possible. If it is not possible for you to resubmit within 3 months, the option to submit a revision will expire, and we would then consider your revised paper as a new submission. However, if you need a little more time, please get in touch and we can extend this deadline for you.

Once again, thank you for submitting your manuscript to Brain Communications and I look forward to receiving your revision.

Yours sincerely,

Dr. Grega Repovs
Associate Editor, Brain Communications

Prof Tara Spires-Jones
Editor in Chief, Brain Communications

Reviewer(s)' Comments to Author:

Reviewer: 1

Comments to the Author
The present study demonstrates that transcutaneous (auricular) stimulation in humans drive reinforcement learning-like effects in human subjects exposed to forced-choice reward-based paradigm. Vagal stimulations followed a cross-sectional, within-subject study design. Overall accuracy was assessed using non-parametric cluster-based permutation tests. The sub-components of the task were modeled using drift-diffusion modeling. The authors report higher accuracies upon vagal stimulation compared to sham stimulation, including stimulation-dependent increases in reward sensitivity and greater accuracies during extinction phases. The authors concluded that their work provides the first evidence for vagal regulation of human reinforcement learning, which might have clinical implications.

This is an interesting study that is in line with animal studies and may have relevant clinical applications. Overall a potentially important contribution. However there are a number of limitations regarding the clarity of the methods that need to be clarified.

One critical aspect that is unclear refers to how exactly the sham condition was defined. Did the subjects detect any sensations upon vagal stimulation? How did the authors reassure themselves that subjects could not discriminate between sham vs vagal conditions? Please explain.

More generally did the subjects report any detectable changes in mood or physiological variables such as gastrointestinal or cardiac tone? The "reward" effect was purely task-dependent?

Another important point that requires clarification is the choice of non-parametric cluster analyses; the manuscript should also include a more in-depth description, preferably not excessively technical, of drift-diffusion modeling. In general the data presented is based on model fitting and would be more clarifying to see more of the raw data associated with the subjects' behaviors.

Reviewer: 2 (Wenfei Han)

Comments to the Author
This is an interesting study where it is shown that auricular vagal stimulation in humans promotes reinforcement learning in a forced choice type of task. The authors report what seem to be clear effects on performance produced by stimulation especially upon weakening (extinction) if reward feedback compared to controls. The results may provide novel ways to interpret the potential clinical implications of auricular vagal stimulation.

However my stimulation was lessened by the lack of details regarding what would be the control condition. It is unclear if subjects were aware of stimulation and if so how this was controlled for. Just the perception of sensory stimulation may have driven subjects to focus and improve performance in response to the experimental setting.

Also how about self reports on subjective feelings such as changes in mood, gastrointestinal sensations, and so on? I believe that without these basic type of information it is difficult to assess the potential quality of the manuscript.

Editorial Office Comments:
- For each statistical test, please report in the figure legend, the N, test used, relevant test statistic, and exact p values (for example F or t values for ANOVAs and t-tests)
- To comply with our policies for rigour and reproducibility, please provide details in each figure legend of the experimental unit (N= trials, subjects). Please note our policy on pseudoreplication, we do not accept analyses where individual trials are the experimental unit when there is likely biological variability is between subjects. Please see instructions for authors for more details. For this type of data, we require either that a mean/median be taken per subject followed by statistical analyses or that more sophisticated analyses are performed such as mixed effects modelling which can account for random effects.
-Please provide details of the statistical tests in the figure legends.
-Please supply an abbreviated summary of up to 50 words (no abbreviations except gene names). This will be used in the table of contents alongside the graphical abstract. Please upload as a separate word document starting with "First author et al…."
