# Supplementary material for: Trust your gut: vagal nerve stimulation in humans improves reinforcement learning
Source: Brain Commun. 2021 Mar 14;3(2):fcab039. doi: 10.1093/braincomms/fcab039 (PMC8066886; doi:10.1093/braincomms/fcab039)
Supplement: fcab039_Supplementary_Data [file fcab039_supplementary_data.zip › Review_files.zip!/Original Submission.pdf]

OXFORD UNIVERSITY PRESS | Brain Communications

Trust your gut: vagal nerve stimulation in humans improves reinforcement learning

Journal:	*Brain Communications*
Manuscript ID	BRAINCOM-2020-251
Manuscript Type:	Original Article
Date Submitted by the Author:	24-Aug-2020
Complete List of Authors:	Weber, Immo; Philipps-Universität Marburg, Department of Neurology<br>Niehaus, Hauke; Philipps-Universität Marburg, Faculty of Psychology, Neuropsychology Section; Philipps-Universität Marburg, Faculty of Psychology, Theoretical Neuroscience Section<br>Krause, Kristina; Philipps-Universität Marburg, Department of Neurology; Philipps-Universität Marburg, Center for Mind, Brain and Behavior (CMBB); Philipps-Universität Marburg, Department of Neurology, Epilepsy Center Hessen<br>Molitor, Lena; Philipps-Universität Marburg, Department of Neurology<br>Peper, Martin; Philipps-Universität Marburg, Faculty of Psychology, Neuropsychology Section; Philipps-Universität Marburg, Center for Mind, Brain and Behavior (CMBB)<br>Schmidt, Laura; Philipps-Universität Marburg, Department of Neurology<br>Hakel, Lukas; Philipps-Universität Marburg, Department of Neurology<br>Timmermann, Lars; Philipps-Universität Marburg, Department of Neurology; Philipps-Universität Marburg, Center for Mind, Brain and Behavior (CMBB)<br>Menzler, Katja; Philipps-Universität Marburg, Department of Neurology; Philipps-Universität Marburg, Center for Mind, Brain and Behavior (CMBB); Philipps-Universität Marburg, Department of Neurology, Epilepsy Center Hessen<br>Knake, Susanne; Philipps-Universität Marburg, Department of Neurology; Philipps-Universität Marburg, Center for Mind, Brain and Behavior (CMBB); Philipps-Universität Marburg, Department of Neurology, Epilepsy Center Hessen<br>Oehrn, Carina; Philipps-Universität Marburg, Department of Neurology; Philipps-Universität Marburg, Center for Mind, Brain and Behavior (CMBB); Philipps-Universität Marburg, Department of Neurology, Epilepsy Center Hessen
Keywords:	

SCHOLARONE™
Manuscripts

Trust your gut: vagal nerve stimulation in humans improves reinforcement learning

Immo Weber[1], Hauke Niehaus[2,3], Kristina Krause[1,4,5], Lena Molitor[1], Martin Peper[2,4], Laura Schmidt[1], Lukas Hakel[1], Lars Timmermann[1,4], Katja Menzler[1,4,5], Susanne Knake[1,4,5], Carina R. Oehrn[1,4,5]

[1] *Department of Neurology, Philipps-University Marburg, Marburg, Germany*

[2] *Faculty of Psychology, Neuropsychology Section, Philipps-University Marburg, Marburg, Germany*

[3] *Faculty of Psychology, Theoretical Neuroscience Section, Philipps-University Marburg, Marburg, Germany*

[4] *Center for Mind, Brain and Behavior (CMBB), Philipps-University Marburg, Marburg, Germany*

[5] *Department of Neurology, Epilepsy Center Hessen, Philipps University, Marburg, Germany*

For Review Only

Correspondence to

Dr. Immo Weber

Department of Neurology

University Hospital Giessen and Marburg

Baldingerstrasse

35043 Marburg

Germany

Phone: +49-6421 58 66496

Email: immo.weber@staff.uni-marburg.de

Abstract

Whereas the effect of vagal nerve stimulation (VNS) on emotional states is well established, its effect on cognitive functions is still unclear. Recent rodent studies show that vagal activation enhances reinforcement learning and neuronal dopamine release. The influence of VNS on reinforcement learning in humans is still unknown. Here, we studied the effect of transcutaneous VNS on reinforcement learning in eight long-standing seizure-free epilepsy patients, using a well-established forced-choice reward-based paradigm in a cross-sectional, within-subject study design. We investigated VNS effects on overall accuracy using non-parametric cluster-based permutation tests. Further, we modelled sub-components of the decision process using drift-diffusion modelling. We found higher accuracies in the VNS condition compared to sham stimulation. Modelling suggests a stimulation-dependent increase of reward sensitivity and shift of accuracy-speed trade-offs towards maximizing rewards. Moreover, VNS was associated with increased non-decision times suggesting enhanced sensory or attentional processes. No differences of starting bias were detected for both conditions. Accuracies in the extinction phase were higher in later trials of the VNS condition suggesting a perseverative effect compared to sham. Together, our results provide first evidence of causal vagal influence on human reinforcement learning and might have clinical implications for usage of vagal stimulation in learning deficiency.

Keywords: vagal nerve stimulation, reinforcement learning, extinction, cognition, decision-making

For Review Only

1. Introduction

For decades, the vagus nerve has been described as the core of the gut-brain axis mediating motivational and emotional states by relaying e.g. food-related information (Breit *et al.*, 2018). In humans, invasive and non-invasive, i.e. transcutaneous, vagal nerve stimulation (VNS) is a common treatment for medication-resistant epilepsy and can thus be used to study causal relationships between the autonomic and the central nervous system (Vonck *et al.*, 2014). While there is a large body of evidence for positive effects of vagal nerve stimulation on anxiety and depression (for a review see Breit *et al.*, 2018), empirical evidence suggesting a relationship between vagal nerve activity and cognitive functions is sparse. Many studies assessed effects of VNS on global measures of cognition in severely affected epilepsy patients and thus blended different cognitive functions (for a review see Vonck *et al.*, 2014). Other studies with Alzheimer's and major depression patients found slight or no effects of VNS on overall cognitive function (Merrill *et al.*, 2006; Sackeim *et al.*, 2001). The few studies that investigated VNS effects on distinct cognitive processes in epilepsy patients and healthy participants did not reveal effects on working memory (Sun *et al.*, 2017), implicit learning (Jongkees *et al.*, 2018) and conflict processing (Fischer *et al.*, 2018). However, Clark and colleagues found positive effects on long-term memory formation when applying VNS during the consolidation phase of a word recognition paradigm in five epilepsy patients (Clark *et al.*, 1999). A large body of evidence from animal and human studies indicates that reward processing is associated with enhanced dopamine release in the brain (for review see Schultz, 2016). Further, rewarding stimulus properties lead to enhanced memory formation through interactions between dopaminergic and mnemonic brain regions (e.g. Dalley and Everitt, 2009).

A recent study in rats demonstrated that optical stimulation of gut-innervating vagal sensory ganglions was associated with increased dopamine release from the substantia nigra and led to enhanced learning of flavor and place preferences and sustained self-stimulation behavior (Han

For Review Only

et al., 2018). This is in line with rodent studies demonstrating enhanced cortical dopamine levels after chronic VNS (Manta *et al.*, 2013) and a cessation of food-related dopamine release in the midbrain after lesions of the hepatic branch of the vagus nerve (Fernandes *et al.*, 2020). These results from animal studies suggest that vagal neurons constitute an essential component of the reward neuronal pathway. A recent study in humans indicates that healthy participants work harder for rewards during VNS compared to sham stimulation (Neuser *et al.*, 2020). The effect of vagal nerve stimulation on reinforcement learning in humans is still unknown. Reinforcement learning involves decision processes, which can be dissected into cognitive sub-processes using drift-diffusion models (DDM, Pedersen *et al.*, 2017). DDM model decision processes based on two performance parameters, i.e. accuracy and reaction time, and constitutes one of the most widely used methods for the investigation of value-based choices (Pedersen *et al.*, 2017). DDM disentangles decision-making into four sub-processes: 1) non-decision operations reflecting perceptual and motor computations 2) internal starting bias towards response options (e.g. elicited by salience) and 3) two core parts of the decision process: i) the drift rate: i.e. how fast the participant extracts relevant stimulus information based on previous experience and ii) boundary separation: how much information is needed in order to make a decision.

Here, we investigate a causal relationship between vagal tone and reinforcement learning in eight long-standing seizure-free, VNS-naive epilepsy patients. In a within-subject design, we applied VNS and sham stimulation while patients performed a reward-based forced choice learning paradigm (Frank *et al.*, 2004). We applied a computerized task, which allowed us to observe effects of VNS on a trial-by-trial basis and to model behavioural sub-components of the decision process using DDM (Pedersen *et al.*, 2017) Corresponding to the animal studies (Han *et al.*, 2018; Manta *et al.*, 2013), we hypothesized that VNS will improve reinforcement learning and lead to a perseverance of learned behaviour.

For Review Only

2. Methods

2.1. Patients

Seventeen patients with temporal lobe epilepsy were recruited from the Department of Neurology at the University Hospital Marburg, Germany. We excluded patients with psychiatric comorbidities by means of the Beck Depression Inventory (BDI-II, Hautzinger *et al.*, 2009) and the Quality of Life in Epilepsy questionnaire (QoLiE, May *et al.*, 2001). As this experiment was part of a larger study investigating VNS effects on neuroimmunological, salimetric and neuropsychological measures, nine patients dropped out on one testing day due to the extensive testing pipeline. Eight patients completed the paradigm on both days (six females, five with left hemisphere epilepsy, mean age±SD: 43.88±10.93 years). Patients had been seizure-free for at least one year (mean±SD 7.25±4.50 years) and completed a neuropsychological test battery including measures of executive functioning, memory, recall and implicit memory. Further, we obtained the NEO Personality Inventory by Costa und McCrae (NEO-PI), assessing the big five personality traits (Ostendorf and Angleitner, 2004) (Supplementary Tab. S1). All patients signed written informed consent. The study was approved by the ethics committee of the University of Marburg and was conducted according to the declaration of Helsinki.

For Review Only

2.2. Paradigm

During stimulation, patients performed a probabilistic forced-choice learning task, in which they could earn money for correct choices (Frank *et al.*, 2004, Fig. 1A). In a subsequent extinction phase, reward was omitted. Patients were seated in an acoustically shielded chamber and performed the task using a standard keyboard in front of a computer monitor. Patients received written and oral instructions and performed several practise trials. Different stimuli

were used for stimulation conditions and instructions. The experimenter ensured that each patient understood the task before starting with the paradigm.

2.3. Transcutaneous vagus nerve stimulation

In a double-blind design, patients received transcutaneous vagus nerve and sham stimulation on the left ear on two separate days - separated by at least two weeks - in pseudo-randomized order (Cerbomed, Nemos, Cerbomed GmbH, Erlangen, Fig. 1B). Patients were continuously stimulated for three hours before and during the paradigm. Stimulation amplitude was set below the average of individual sensitivity and pain threshold of each patient (mean±SD stimulation amplitude: tVNS 1.29±0.61 mA; sham: 1.25±0.49 mA).

For Review Only

2.4. Statistics

The identical analyses were performed for the study and extinction phase. We performed all calculations with Matlab 2016b (The Mathworks inc), the DMAT toolbox (Vandekerckhove and Tuerlinckx, 2008), the Fieldtrip toolbox (Oostenveld *et al.*, 2011) and self-written code. We set the alpha level to 0.05.

Effects on accuracy

To analyse the time-resolved update of accuracies, we calculated the cumulative accuracies per trial according to:

$$cACC(trial) = \frac{\sum_{i=1}^{trial} correct\ trials}{trial}, \qquad (1)$$

We evaluated differences between the VNS and sham condition by means of non-parametric cluster-based permutation tests (Maris and Oostenveld, 2007). This procedure ranks effect sizes in relation to a permutation distribution effectively controlling for multiple comparisons. The surrogate distribution was created by randomising condition labels (10.000 permutations).

Drift-diffusion modelling

We modelled components of the decision process using DDM for continuous non-overlapping blocks of 30 trials using the DMAT-toolbox (Vandekerckhove and Tuerlinckx, 2008; Supplementary Fig. S1). First, we fitted a model, in which parameters were allowed to change freely between stimulation conditions. Second, we compared the evidence to a model with fixed parameters for both conditions using a Chi-square test, to test if any difference between stimulation conditions could be detected. If the models differed significantly, we performed a second level analysis, to disentangle for which parameters the stimulation effect occurs. For this, we fixed all but one parameter and repeated the previous testing for all four parameters. All statistical comparisons were Bonferroni corrected.

For Review Only

Prediction of stimulation effect on accuracy

In order to assess the impact of patient characteristics and disease-related factors on the stimulation effect, we estimated the mutual information quotient (MIQ) between the mean accuracy differences between the stimulation conditions in the significant time window and potential predictors (i.e. age, sex, NEO-PI scores and disease-related information (Gulgezen *et al.*, 2009). The MIQ is particularly suited for the estimation of shared information between two variables in small samples while accounting for redundant information between all predictors. We used a binning estimator and adapted bin-sizes according to the Freedman-Diaconis rule

(Freedman and Diaconis, 1981). We determined significant MIQ estimates by means of a surrogate-based permutation test, where labels of response and predictor variables were randomly swapped (10.000 permutations). P-values were subsequently corrected for multiple comparisons using Bonferroni correction.

2.5. Data availability

All data and code used for analysis are available upon reasonable request.

For Review Only

3. Results

3.1. Learning phase

Effect of stimulation on performance

Cluster statistics revealed that accuracies were significantly larger in the VNS condition than in the sham condition, which was time-specific (trial 123-240, $p=0.04$, Fig. 2A, Supplementary Fig. S2). Subsequent tests against chance level demonstrated that patients effectively learned in the VNS (trials 26-240: $p<0.01$), but not in the sham condition (no cluster found). There was no effect of stimulation order (day 1 vs. day 2) on accuracies (largest cluster: $p=0.20$). We did not find stimulation effects on reaction times (largest cluster: $p=0.33$, for individual reaction times see supplementary Tab. S1).

Drift and diffusion model

Each of the eight most liberal DDM-models for each trial block was significantly different from models with fixed parameters for both simulation conditions (all $p<0.001$, for fit values see Supplementary Tab. S2). A second-level analysis revealed a relatively higher non-decision time (all $p<0.028$, block 3-7, Fig. 2B) boundary separation (all $p<0.017$, block 3-7, Fig. 2C) and drift

rate (all $p<0.013$, Fig. 2D) in the VNS condition. There were no effects of stimulation on starting bias (all $p>0.11$, Fig. 2E).

Prediction of stimulation effect

The analysis of MIQ revealed that the NEO-PI sub score extraversion significantly shared predictive information with the stimulation effect (MIQ=3.90, $p<0.001$, Fig. 3). None of the other parameters predicted the stimulation effected (all $p> 0.11$).

For Review Only

3.2. Extinction phase

Cluster statistics showed that accuracies were higher for VNS in comparison to the sham condition. This effect was specific for later trials of the extinction phase (trials 66-120: p=0.02, Fig. 4A, Supplementary Fig. S3) indicating a perseverative effect of stimulation on learning. There was no effect of stimulation order on accuracy (no cluster found) or reaction times (no cluster found).

Drift and diffusion model

Blocks one, three and four of the most liberal DDM-models yielded significant differences between stimulation conditions (Block 1: p=0.01, Block 2: p=0.25, Block 3: $p<0.001$, Block 4: $p<0.01$, fit values: see Supplementary Tab. S3). Analogous to the learning phase, we found stimulation effects on non-decision time (p=0.02, block 4, Fig. 4B), boundary separation (p=0.04, block 1, Fig. 4C) and drift-rate ($p<0.01$, block 4, Fig. 4D). There was no effect on starting bias (all $p>0.20$, Fig. 4E).

4. Discussion

In this study, we show for the first time that VNS in humans positively influences reinforcement learning. During the learning phase, VNS was associated with a higher overall accuracy of decisions (Fig. 2A) than during sham stimulation. The effect was time-specific, i.e. it appeared after several trials of learning (123 trials). After omission of reward, VNS led to a perseverance of learned behaviour. This effect occurred in the second half of the extinction phase, when accuracy had dropped during sham stimulation, but not VNS.

These results are in accordance with a previous study in rats demonstrating that optical stimulation of vagal sensory neurons was associated with enhanced reinforcement learning and perseverance of behavior, analogous to the decelerated extinction in our study (Han *et al.*, 2018). Along with other results in rats (Manta *et al.*, 2013), this rodent study indicates that vagal stimulation is associated with enhanced dopamine release in the brain. It is well established that dopamine release encodes prediction errors – the discrepancy between expected and observed reward – and is the core neurotransmitter involved in reinforcement learning (for review see Schultz, 2016). Thus, one could speculate that enhanced dopamine release by vagal stimulation constitutes the underlying neural mechanism of the effects of VNS on reinforcement learning observed in this study. However, here, we do not explicitly demonstrate the role of dopamine and it is very likely that VNS additionally activates other transmitter systems. Several studies demonstrated that VNS increases serotonin in the animal (Manta *et al.*, 2013) and norepinephrine levels in the human brain (Ventura-Bort *et al.*, 2018), both of which are associated with reward expectation (Yi Li *et al.*, 2016; Sadacca *et al.*, 2017).

Further, we investigated the effects of VNS on sub-components of reward-based decision-making. We found effects of VNS on boundary separation and drift rates (Fig. 2C-D). In reward-based paradigms, drift rate can be interpreted as a proxy of reward sensitivity (Pedersen *et al.*, 2017). Additionally, VNS raised the internal decision threshold, favouring accuracy over

For Review Only

speed in comparison to sham stimulation (boundary separation, Fig. 2C). Based on the assumption that vagal stimulation promotes dopaminergic activity (Han *et al.*, 2018), our results are in line with previous studies demonstrating a positive correlation of dopaminergic activity and reward sensitivity in animal (e.g. Tobler *et al.*, 2005) and human studies (e.g. Hahn *et al.*, 2011).

We found that VNS during learning did not only enhance processes linked to decision-making, but also increased behavioural components tied to sensory and attention processes (non-decision time, Fig. 2B). This result may be linked to previous reports of increased post-error slowing during vagal stimulation in humans (i.e. slowing of the subsequent response, Sellaro *et al.*, 2015). Other VNS studies reported an increase of attentional processes, which may bind cognitive resources during decision-making (Maraver *et al.*, 2020). This explanation is further supported by effectiveness of dopamine-releasing drugs for patients with attention-deficit disorder (Madras *et al.*, 2005). Stimulation did not affect internal preference for either response (starting bias, Fig. 2E), which demonstrates the specificity of the stimulation effects on the latter three aspects of decision-making.

Mutual information analysis revealed that no patient characteristics or disease-related factors impacted the stimulation effect on reinforcement learning. We found a selective relationship between extraversion of patients and the extent of the stimulation effects. A supplementary analysis indicates that patients with relatively lower extraversion profited most from stimulation, i.e. the stimulation effect in patients deviated during sham, but not VNS stimulation (Supplementary Fig. S3). These results are in accordance with previous studies showing that individual differences in extraversion predict reward-sensitivity in humans (e.g. Cohen *et al.*, 2005). However, this finding should be interpreted with caution due to the small sample size and should be corroborated in future studies.

For Review Only

One limitation of our study is the small simple size. However, we show consistent results across patients. We analysed the data conservatively by using nonparametric statistics and corrected rigorously type I errors were applicable. A second drawback of our study is the study population. Due to ethical reasons, we refrained from applying VNS to healthy participants. However, to approximate effects in the healthy population and make inferences more generalizable, we exclusively included long-standing (i.e. minimum of one year) seizure-free patients without neuropsychological deficits.

In summary, we demonstrate for the first time that vagal nerve stimulation in humans enhances reinforcement learning and decelerates extinction of learned behaviour. Behavioural modelling indicates that vagal tone increases reward sensitivity and shifts accuracy-speed trade-offs towards maximizing rewards.

For Review Only

5. Acknowledgements

The authors would like to thank the patients for participating in this study.

6. Funding

Authors declare no project-related funding.

7. Competing interests

I.W., H.N., K.K., L.M., M.P, L.S., L.H., K.M., S.K., C.R.O. declare no competing interests. L.T. reports grants, personal fees, and non-financial support from SAPIENS Steering Brain Stimulation, Medtronic, Boston Scientific, and St. Jude Medical and has received payments from Bayer Healthcare, UCB Schwarz Pharma, and Archimedes Pharma and also honoraria as

a speaker on symposia sponsored by Teva Pharma, Lundbeck Pharma, Bracco, Gianni PR, Medas Pharma, UCB Schwarz Pharma, Desitin Pharma, Boehringer Ingelheim, GSK, Eumecom, Orion Pharma, Medtronic, Boston Scientific, Cephalon, Abbott, GE Medical, Archimedes, and Bayer.

For Review Only

8. References

Breit S, Kupferberg A, Rogler G, Hasler G. Vagus Nerve as Modulator of the Brain–Gut Axis in Psychiatric and Inflammatory Disorders. Front. Psychiatry 2018; 9.

Clark KB, Naritoku DK, Smith DC, Browning RA, Jensen RA. Enhanced recognition memory following vagus nerve stimulation in human subjects. Nature neuroscience 1999; 2: 94–98.

Cohen MX, Young J, Baek J-M, Kessler C, Ranganath C. Individual differences in extraversion and dopamine genetics predict neural reward responses. Brain research. Cognitive brain research 2005; 25: 851–861.

Dalley JW, Everitt BJ. Dopamine receptors in the learning, memory and drug reward circuitry. Seminars in cell & developmental biology 2009; 20: 403–410.

Fernandes AB, Alves da Silva J, Almeida J, Cui G, Gerfen CR, Costa RM, et al. Postingestive Modulation of Food Seeking Depends on Vagus-Mediated Dopamine Neuron Activity. Neuron 2020; 106: 778-788.e6.

Fischer R, Ventura-Bort C, Hamm A, Weymar M. Transcutaneous vagus nerve stimulation (tVNS) enhances conflict-triggered adjustment of cognitive control. Cogn Affect Behav Neurosci 2018; 18: 680–693.

Frank MJ, Seeberger LC, O'reilly RC. By carrot or by stick: cognitive reinforcement learning in parkinsonism. Science (New York, N.Y.) 2004; 306: 1940–1943.

Freedman D, Diaconis P. On the histogram as a density estimator:L 2 theory. Z. Wahrscheinlichkeitstheorie verw Gebiete 1981; 57: 453–476.

Gulgezen G, Cataltepe Z, Yu L. Stable and Accurate Feature Selection. In: Buntine W, Grobelnik M, Mladenić D, Shawe-Taylor J, editors. Machine learning and knowledge discovery in databases. European conference, ECML PKDD 2009, Bled, Slovenia, September 7 - 11, 2009 ; proceedings, part I. Berlin: Springer; 2009. p. 455-68.

Hahn T, Heinzel S, Dresler T, Plichta MM, Renner TJ, Markulin F, et al. Association between reward-related activation in the ventral striatum and trait reward sensitivity is moderated by dopamine transporter genotype. Human brain mapping 2011; 32: 1557–1565.

Han W, Tellez LA, Perkins MH, Perez IO, Qu T, Ferreira J, et al. A Neural Circuit for Gut-Induced Reward. Cell 2018; 175: 665-678.e23.

For Review Only

Hautzinger M, Keller F, Kühner C. Beck Depressions-Inventar.- Manual. BDI II; 2009.

Jongkees BJ, Immink MA, Finisguerra A, Colzato LS. Transcutaneous Vagus Nerve Stimulation (tVNS) Enhances Response Selection During Sequential Action. Frontiers in psychology 2018; 9: 1159.

Madras BK, Miller GM, Fischman AJ. The dopamine transporter and attention-deficit/hyperactivity disorder. Biological psychiatry 2005; 57: 1397–1409.

Manta S, El Mansari M, Debonnel G, Blier P. Electrophysiological and neurochemical effects of long-term vagus nerve stimulation on the rat monoaminergic systems. The international journal of neuropsychopharmacology 2013; 16: 459–470.

Maraver MJ, Steenbergen L, Hossein R, Actis-Grosso R, Ricciardelli P, Hommel B, et al. Transcutaneous vagus nerve stimulation modulates attentional resource deployment towards social cues. Neuropsychologia 2020; 143: 107465.

Maris E, Oostenveld R. Nonparametric statistical testing of EEG- and MEG-data. Journal of neuroscience methods 2007; 164: 177–190.

May TW, Pfäfflin M, Cramer JA. Psychometric Properties of the German Translation of the QOLIE-31. Epilepsy & behavior : E&B 2001; 2: 106–114.

Merrill CA, Jonsson MAG, Minthon L, Ejnell H, C-son Silander H, Blennow K, et al. Vagus nerve stimulation in patients with Alzheimer's disease: Additional follow-up results of a pilot study through 1 year. The Journal of clinical psychiatry 2006; 67: 1171–1178.

Neuser MP, Teckentrup V, Kühnel A, Hallschmid M, Walter M, Kroemer NB. Vagus nerve stimulation boosts the drive to work for rewards. Nature communications 2020; 11: 3555.

Oostenveld R, Fries P, Maris E, Schoffelen J-M. FieldTrip: Open source software for advanced analysis of MEG, EEG, and invasive electrophysiological data. Computational intelligence and neuroscience 2011; 2011: 156869.

Ostendorf F, Angleitner A. NEO-PI-R. NEO-Persönlichkeitsinventar nach Costa und McCrae. Göttingen, Bern, Toronto, Seattle: Hogrefe Verlag für Psychologe; 2004.

Pedersen ML, Frank MJ, Biele G. The drift diffusion model as the choice rule in reinforcement learning. Psychonomic bulletin & review 2017; 24: 1234–1251.

Sackeim HA, Keilp JG, Rush AJ, George MS, Marangell LB, Dormer JS, et al. The effects of vagus nerve stimulation on cognitive performance in patients with treatment-resistant depression. Neuropsychiatry, neuropsychology, and behavioral neurology 2001; 14: 53–62.

For Review Only

Sadacca BF, Wikenheiser AM, Schoenbaum G. Toward a theoretical role for tonic norepinephrine in the orbitofrontal cortex in facilitating flexible learning. Neuroscience 2017; 345: 124–129.

Schultz W. Dopamine reward prediction-error signalling: a two-component response. Nature reviews. Neuroscience 2016; 17: 183–195.

Sellaro R, van Leusden JWR, Tona K-D, Verkuil B, Nieuwenhuis S, Colzato LS. Transcutaneous Vagus Nerve Stimulation Enhances Post-error Slowing. Journal of cognitive neuroscience 2015; 27: 2126–2132.

Sun L, Peräkylä J, Holm K, Haapasalo J, Lehtimäki K, Ogawa KH, et al. Vagus nerve stimulation improves working memory performance. Journal of clinical and experimental neuropsychology 2017; 39: 954–964.

Tobler PN, Fiorillo CD, Schultz W. Adaptive coding of reward value by dopamine neurons. Science (New York, N.Y.) 2005; 307: 1642–1645.

Vandekerckhove J, Tuerlinckx F. Diffusion model analysis with MATLAB: a DMAT primer. Behavior research methods 2008; 40: 61–72.

Ventura-Bort C, Wirkner J, Genheimer H, Wendt J, Hamm AO, Weymar M. Effects of Transcutaneous Vagus Nerve Stimulation (tVNS) on the P300 and Alpha-Amylase Level: A Pilot Study. Frontiers in human neuroscience 2018; 12: 202.

Vonck K, Raedt R, Naulaerts J, Vogelaere F de, Thiery E, van Roost D, et al. Vagus nerve stimulation…25 years later! What do we know about the effects on cognition? Neuroscience & Biobehavioral Reviews 2014; 45: 63–71.

Yi Li, Weixin Zhong, Daqing Wang, Qiru Feng, Zhixiang Liu, Jingfeng Zhou, et al. Serotonin neurons in the dorsal raphe nucleus encode reward signals. Nat Commun 2016; 7: 1–15.

For Review Only

9. Figure captions

Fig. 1: Experimental design and electrode placement. **A)** The task comprised two consecutive parts: the learning and the extinction phase. During the learning phase, patients were repeatedly presented with three fixed pairs of stimuli (240 trials, 80 trials per set). The stimuli consisted of six letters from the artificial Klingon alphabet (https://www.dafont.com), which were organized in three sets. Set A consisted of one stimulus with a reward probability of 80 % and one with 20 %, set B with 70 % and 30 % and set C with 60 % and 40 %, respectively. Patients were instructed to indicate as fast as possible by button press, which of both stimuli was associated with a higher reward. Pairs during the learning phase were fixed, but the side of presentation randomized for each trial. Associations between stimuli and reward probabilities were randomized for each patient. For each testing day, a different set of stimuli was used. After completion of the learning phase, an extinction phase followed, in which no feedback was given. During this phase, in each trial, a pseudo-random combination of the six previously presented letters was presented (120 trials). In total, the duration of the paradigm was approximately 15 min. **B)** An independent clinician attached the stimulation device and concealed it with a headband covering the entire ear. In the sham condition, the probe was applied to the center of the left lobule (top). In the active VNS condition, the probe was applied to the left cymba conchae in order to stimulate the auricular branch of the vagus nerve according to the guidelines of the manufacturer (bottom, image from Jongkees *et al.*, 2018). Patients were told that the aim of the study was to compare the effectiveness of different stimulation sites for VNS without priming patients to expected effects at each probe position.

For Review Only

Fig. 2: Effect of stimulation on reinforcement learning (learning phase). A) Cluster-based permutation statistic revealed higher accuracy during the VNS compared to the sham condition (trials: 123-240, $p=0.04$). Coloured areas indicate standard errors. The dotted line indicates chance level at 50%. **B-E)** We found time (i.e. trial) – specific effects of stimulation on DDM parameters during the learning phase (Chi-square test, Bonferroni corrected): **B:** non-decision time ($p<0.03$), **C:** starting bias ($p>0.11$), **D:** drift rate ($p<0.01$), **E:** Boundary separation ($p<0.02$). In all subplots, shaded grey areas indicate significant differences between stimulation conditions.

For Review Only

Fig. 3: Predictor importance of patient variables on stimulation effect (MIQ analysis). The individual extraversion scores of patients share most predictive information with the stimulation effect on accuracy: ***=$p<0.01$ (non-parametric permutation test, Bonferroni corrected). MIQ: mutual information quotient.

For Review Only

Fig. 4: Effect of stimulation on accuracy in extinction phase. **A)** Cluster-based permutation statistic shows differences between stimulation conditions on accuracy (trials 66-120, p=0.02). Coloured areas indicate standard errors. The dotted line indicates chance level at 50%. **B-E**) We found time (i.e. trial) – specific effects of stimulation on DDM parameters during the extinction phase (Chi-square test, Bonferroni corrected): **B:** non-decision time (p=0.02), **C**: starting bias (p>0.20), **D:** drift rate (p<0.01), **E:** boundary separation (p=0.04). In all subplots, grey shaded areas indicate significant differences between conditions.

For Review Only

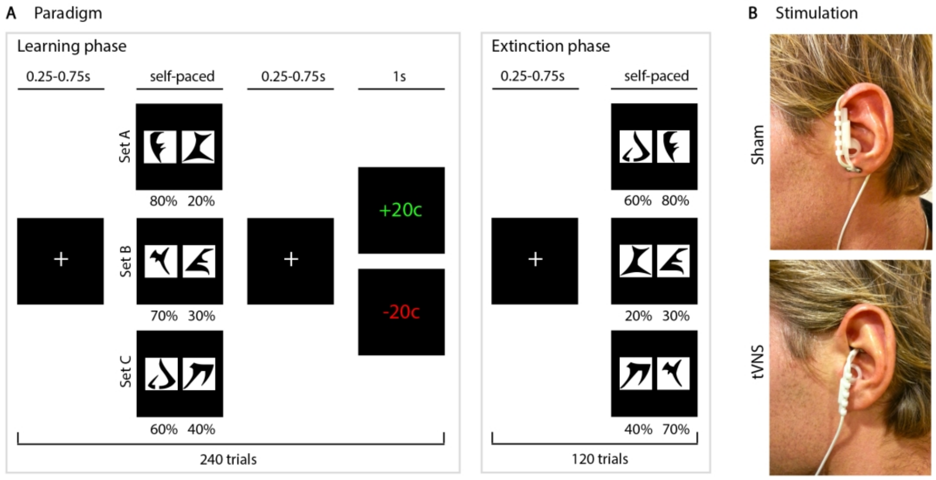

Fig. 1: Experimental design and electrode placement. A) The task comprised two consecutive parts: the learning and the extinction phase. During the learning phase, patients were repeatedly presented with three fixed pairs of stimuli (240 trials, 80 trials per set). The stimuli consisted of six letters from the artificial Klingon alphabet (https://www.dafont.com), which were organized in three sets. Set A consisted of one stimulus with a reward probability of 80 % and one with 20 %, set B with 70 % and 30 % and set C with 60 % and 40 %, respectively. Patients were instructed to indicate as fast as possible by button press, which of both stimuli was associated with a higher reward. Pairs during the learning phase were fixed, but the side of presentation randomized for each trial. Associations between stimuli and reward probabilities were randomized for each patient. For each testing day, a different set of stimuli was used. After completion of the learning phase, an extinction phase followed, in which no feedback was given. During this phase, in each trial, a pseudo-random combination of the six previously presented letters was presented (120 trials). In total, the duration of the paradigm was approximately 15 min. B) An independent clinician attached the stimulation device and concealed it with a headband covering the entire ear. In the sham condition, the probe was applied to the center of the left lobule (top). In the active VNS condition, the probe was applied to the left cymba conchae in order to stimulate the auricular branch of the vagus nerve according to the guidelines of the manufacturer (bottom, image from Jongkees et al., 2018). Patients were told that the aim of the study was to compare the effectiveness of different stimulation sites for VNS without priming patients to expected effects at each probe position.

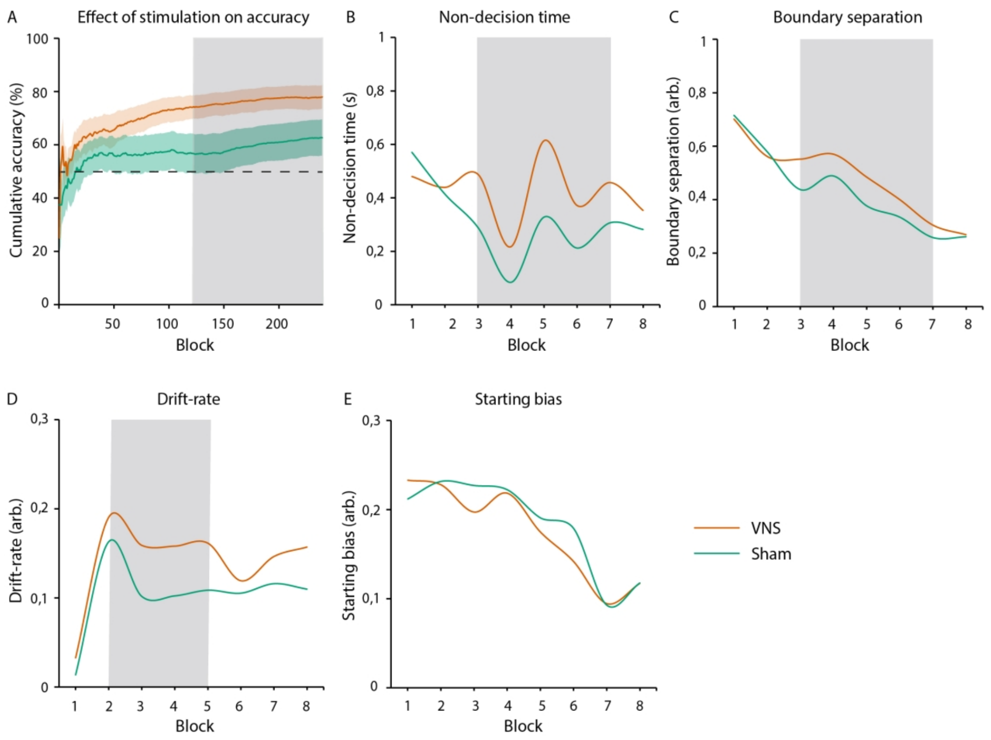

Fig. 2: Effect of stimulation on reinforcement learning (learning phase). A) Cluster-based permutation statistic revealed higher accuracy during the VNS compared to the sham condition (trials: 123-240, p=0.04). Coloured areas indicate standard errors. The dotted line indicates chance level at 50%. B-E) We found time (i.e. trial) – specific effects of stimulation on DDM parameters during the learning phase (Chi-square test, Bonferroni corrected): B: non-decision time (p<0.03), C: starting bias (p>0.11), D: drift rate (p<0.01), E: Boundary separation (p<0.02). In all subplots, shaded grey areas indicate significant differences between stimulation conditions.

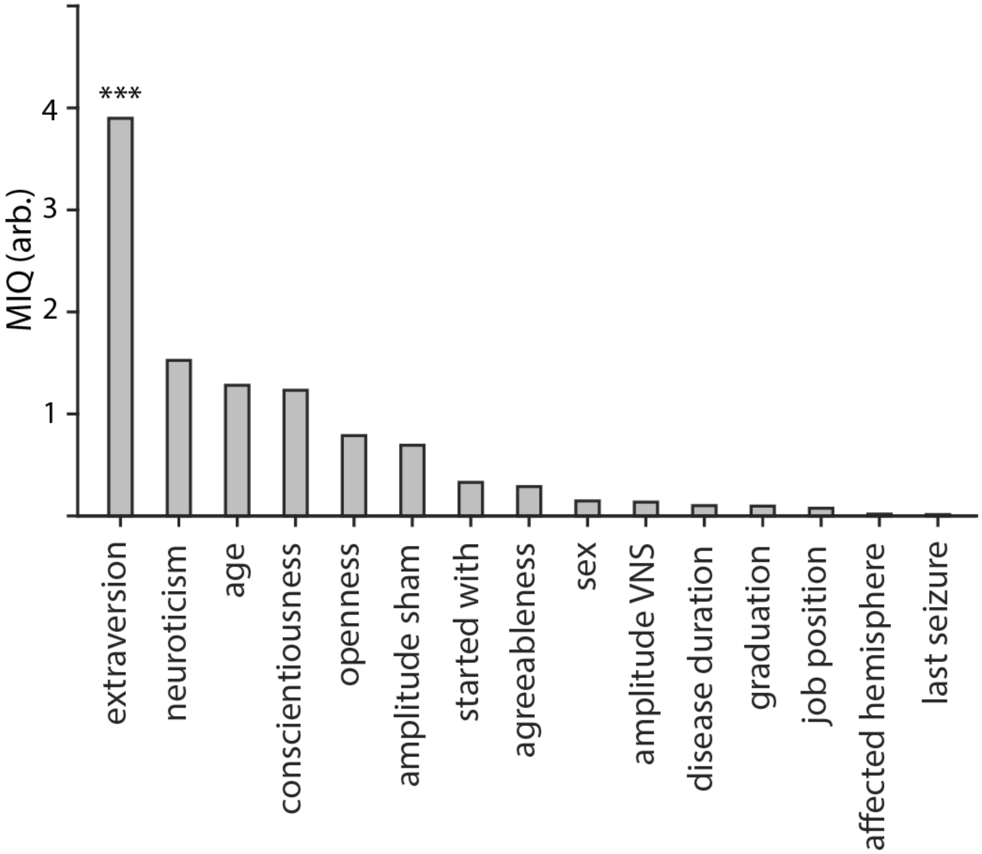

Fig. 3: Predictor importance of patient variables on stimulation effect (MIQ analysis). The individual extraversion scores of patients share most predictive information with the stimulation effect on accuracy: ***=p<0.01 (non-parametric permutation test, Bonferroni corrected). MIQ: mutual information quotient.

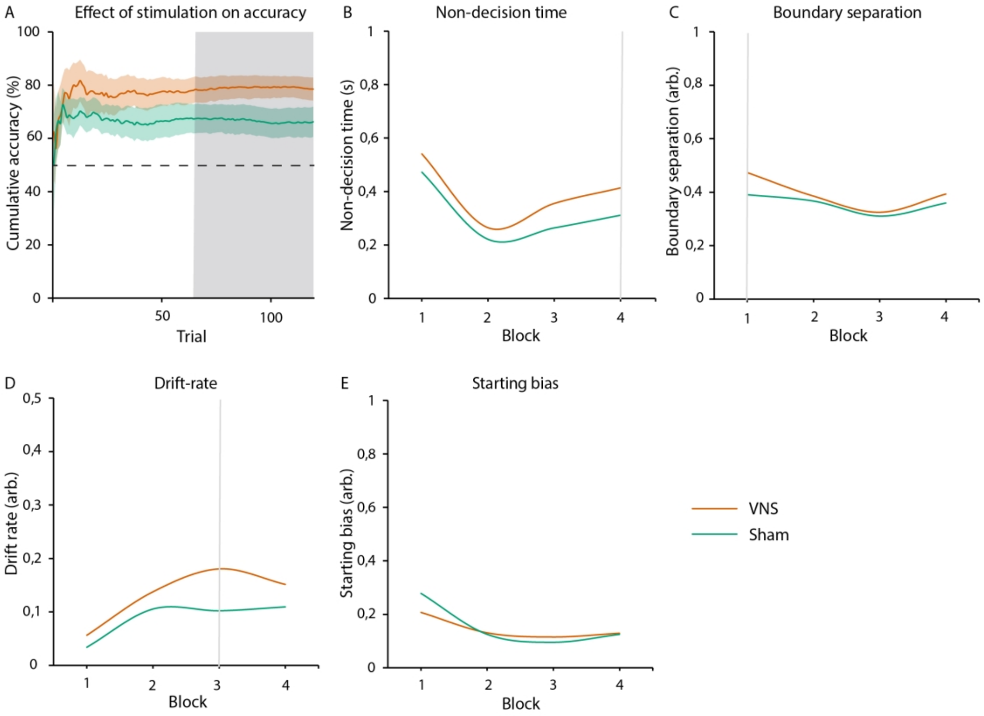

Fig. 4: Effect of stimulation on accuracy in extinction phase. A) Cluster-based permutation statistic shows differences between stimulation conditions on accuracy (trials 66-120, p=0.02). Coloured areas indicate standard errors. The dotted line indicates chance level at 50%. B-E) We found time (i.e. trial) – specific effects of stimulation on DDM parameters during the extinction phase (Chi-square test, Bonferroni corrected): B: non-decision time (p=0.02), C: starting bias (p>0.20), D: drift rate (p<0.01), E: boundary separation (p=0.04). In all subplots, grey shaded areas indicate significant differences between conditions.

Supplementary material

Table S1: Patient data

ID			1	2	3	4	5	6	7	8
Sex			f	m	f	f	f	f	f	m
Age			55	53	53	32	42	41	25	50
Disease duration			42	22	25	17	33	28	9	6
Affected hemisphere			right	left	right	left	left	left	left	right
Years since last seizure (YLS)			1	16	5	9	5	10	5	7
Stimulation amplitude (mA)	VNS		2.5	1.0	0.8	0.9	1.5	1.8	1.0	0.8
	Sham		1.0	0.7	1.2	2.2	0.8	1.7	1.2	1.2
Started with (SW)			Sham	ON	ON	Sham	Sham	ON	ON	Sham
Graduation			1	3	3	3	3	2	3	1
Highest job position			2	4	2	2	3	2	2	1
WAIS-IV	MZ_RW		15	24	20	18	25	22	24	12
	MZ_WP		9	14	10	8	15	11	12	6
Socio-demographic estimation scale	Verbal-IQ (VIQ)		96	126	109	107	122	101	119	94
	Total IQ (TIQ)		99	115	109	109	124	103	118	93
NEO-PI	Neuroticism (Neu)		3.92	3.06	2.77	3.21	2.81	3.02	3.56	3.27
	Extraversion (Ext)		3.13	3.29	2.81	3.46	3.67	3.35	3.25	3.50
	Openness (Ope)		3.79	3.35	2.98	3.48	3.73	3.69	4.04	3.69
	Agreeableness (Agr)		3.58	3.63	3.46	3.94	3.65	3.71	3.71	3.83
	Conscientiousness (Con)		3.42	3.56	3.54	3.52	3.71	3.69	3.96	3.96
Reaction times (s)	Learning phase	VNS	1.79	1.32	1.59	2.20	3.49	1.36	1.47	1.12
		Sham	1.66	1.34	1.69	1.04	2.42	0.81	1.65	1.21
	Extinction phase	VNS	1.64	1.41	1.40	2.08	1.98	1.28	1.50	0.85
		Sham	1.19	0.96	1.54	1.09	1.92	1.20	1.49	1.23

f: female, m: male; graduation: 1=no graduation, 2= secondary school, 3= technical diploma/general higher education entrance qualification; highest job position: 0= no job, 1=trainee, 2= office worker/civil servants, 3= academics/students, 4= senior staff/senior officer, WAIS-IV: Wechsler Adult Intelligence Scale, MZ_RW: raw values of matrix reasoning test, MZ_WP: point values of matrix reasoning test, stimulation amplitude was not different between conditions (p=0.90, two-sided paired t-test).

For Review Only

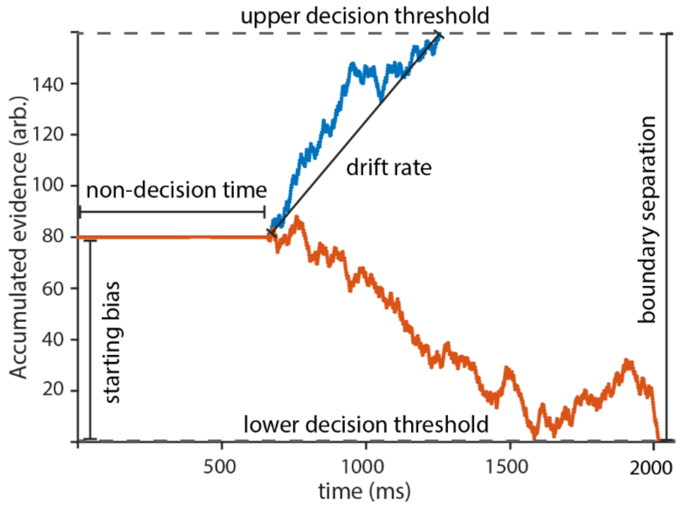

Fig. S1: Schematic representation of the drift and diffusion model. One possibility to model the decision process in a two-alternative forced choice paradigm is using the well-established drift and diffusion model. The DDM assumes that subjects slowly accumulate and integrate evidence at every time-step for one of two choices until a decision boundary is reached (dotted lines). The evidence, which constitutes sensory input and its integration with prior knowledge is assumed to be noisy and thus modelled as a stochastic process. The red and blue line represent the accumulated evidence associated with the two opposing choices for the decision. Using the reaction time of individual subjects, the decision process may be dismantled into four parameters: non-decision time, starting bias, drift rate and boundary separation.

For Review Only

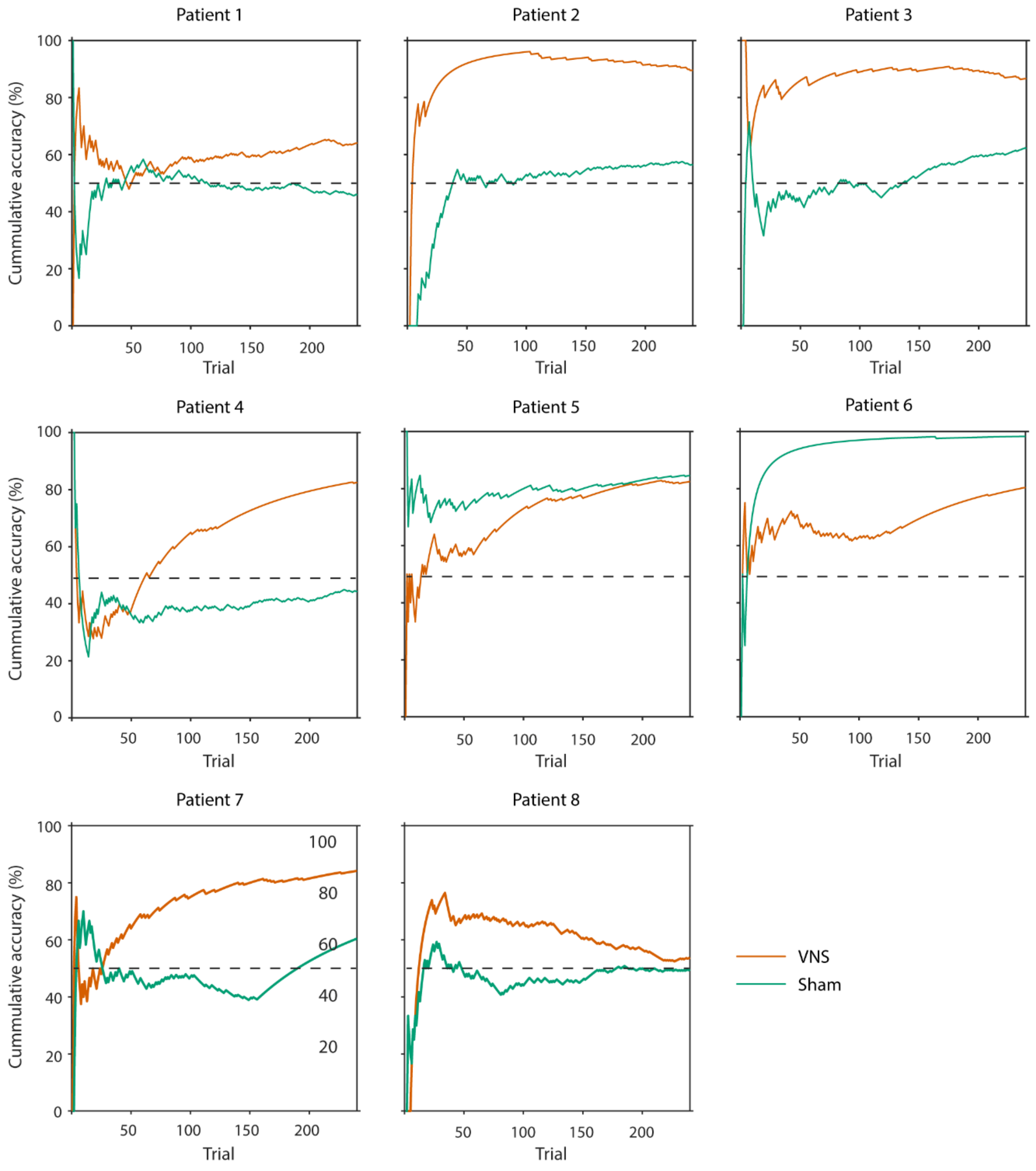

Fig. S2: Individual cumulative accuracies during learning phase for each patient per condition. Dotted lines represent chance level at 50%.

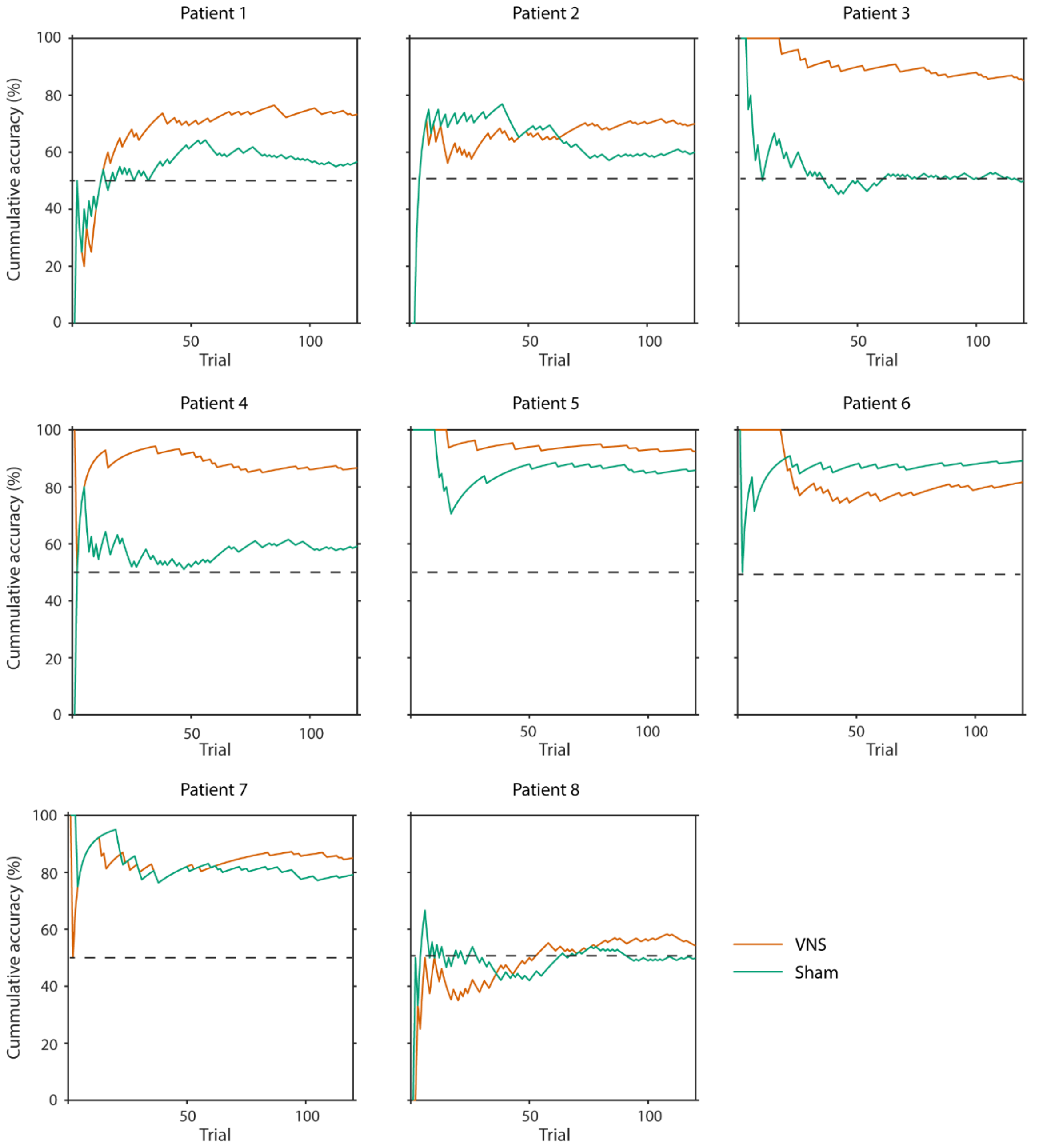

Fig. S3: Individual cumulative accuracies during extinction phase for each patient per condition. Dotted lines represent chance level at 50%.

Table S2: Badness-of-fit values for learning phase

	Phase	Learning							
	Block	1	2	3	4	5	6	7	8
Fitvalue	No stim. Effect	911.51	1079.25	1182.30	1308.13	1353.99	1242.53	1396.77	1469.83
	Stim. Effect	851.51	1031.24	1115.00	1253.46	1278.71	1209.25	1353.08	1445.12

Shown are the badness-of-fit values of the DDMs per trial block during the learning phase. Stim. Effect: all DDM-parameters were allowed to vary during stimulation condition, No stim. Effect: fixed parameters. The lower the values, the better is the modelfit.

Table S3: Badness-of-fit values for extinction phase

	Phase	Extinction			
	Block	1	2	3	4
Fitvalue	No stim. Effect	1137.44	1237.45	1249.21	1328.56
	Stim. Effect	1115.76	1223.98	1215.40	1302.38

Shown are the badness-of-fit values of the DDMs per trial block during the extinction phase. Stim. Effect: all DDM-parameters were allowed to vary during stimulation condition, No stim. Effect: fixed parameters. The lower the values, the better is the model fit.

For Review Only

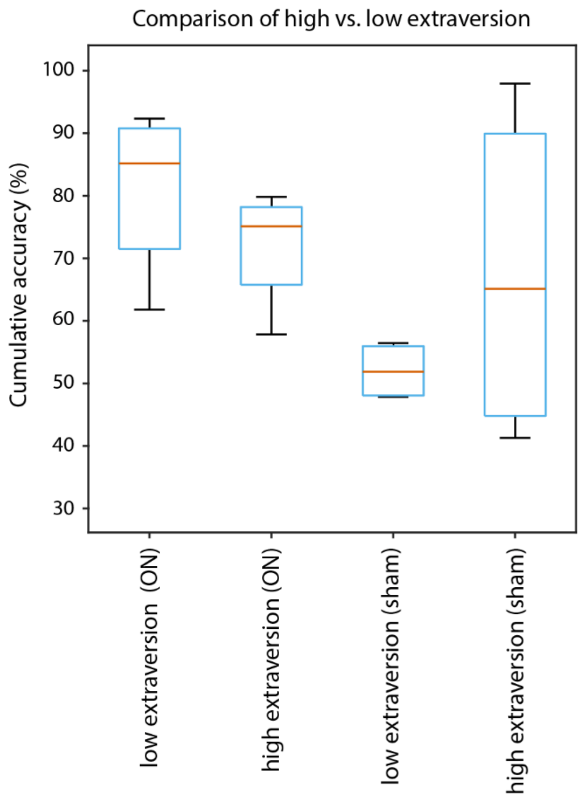

Fig. S4: Relationship of accuracies for low vs. high extraversion and both stimulation conditions (n=4 per group). The upper and lower edges of the boxes indicate the 25th and 75th percentile, respectively. Red lines indicate the median and whiskers the most extreme values.
